# A new subterranean species and an updated checklist of *Strumigenys* (Hymenoptera, Formicidae) from Macao SAR, China, with a key to species of the Greater Bay Area

**DOI:** 10.3897/zookeys.970.54958

**Published:** 2020-09-21

**Authors:** François Brassard, Chi-Man Leong, Hoi-Hou Chan, Benoit Guénard

**Affiliations:** 1 The Insect Biodiversity and Biogeography Laboratory School of Biological Sciences, The University of Hong Kong, Pok Fu Lam Rd, Lung Fu Shan, Hong Kong SAR, China University of Hong Kong Hong Kong China; 2 Division of Nature Conservation Studies, Instituto Para Os Assuntos Municipais, Macao SAR, China Macao Science Center Macao China; 3 Macao Science Center, Avenida Dr. Sun Yat-Sen, Macao SAR, China Instituto Para Os Assuntos Municipais Macao China

**Keywords:** ants, hypogaeic, species list, subterranean, taxonomic key, urban

## Abstract

In the past few decades, sampling of leaf litter with Winkler extractors revealed how abundant and ubiquitous ants from the genus *Strumigenys* are. It is now known that this genus has the third greatest number of species within the Formicidae family. However, very few subterranean species are known, which may be due to the current under-sampling of the soil stratum. Here, a holistic sampling approach, including the use of subterranean traps, was employed in Macao SAR, China. Subterranean traps allowed the collection of a new cryptic ant species: *Strumigenys subterranea* Brassard, Leong & Guénard, **sp. nov.** Moreover, extensive sampling of the leaf litter in secondary forests provided four new species records for the genus. The list of Macanese *Strumigenys* is here updated, elevating the known diversity in Macao to nine species. Furthermore, to facilitate further research on ants of the Guangdong-Hong Kong-Macao Greater Bay Area, a key to the 29 *Strumigenys* species known from the region is provided. Lastly, recommendations for sampling methods to assess ant biodiversity underground are discussed. In conclusion, this study highlights the importance of using extensive sampling methods, and suggests that relatively small patches of secondary forests within cities can hold a surprisingly high diversity as well as some undescribed species.

## Introduction

With a total of 851 described extant species (AntCat.org 2020), *Strumigenys* is one of the most diverse ant genera. Primarily distributed within tropical and subtropical regions, several species occur nonetheless within temperate regions (Janicki et al. 2016; Guénard et al. 2017). Yet, the *Strumigenys* diversity currently reported within tropical regions is likely underestimated. For example, in Southeast Asia, several authors have shown that unrecorded and undescribed *Strumigenys* species should be expected within several countries (e.g., Guénard et al. 2010; Eguchi et al. 2011; Liu et al. 2015; Jaitrong et al. 2016; Tang et al. 2019). Accordingly, recent work sampling leaf-litter ant communities in Yunnan and Hong Kong resulted in a substantial increase in their known *Strumigenys* diversity, both because of unrecorded and undescribed species (Liu et al. 2015; Tang et al. 2019).

Morphologically, *Strumigenys* species are easily distinguished by their small body size, the spongiform tissues on their metasoma (when present), their specialized pilosity and their opposable mandibles (Bolton 1999). Phylogenetically, species of *Strumigenys* were recently moved from the Dacetini to the Attini tribe based on molecular analyses, strengthening their position as the sister taxon to the phalacromyrmecine ants (Ward et al. 2015). Ecologically, *Strumigenys* species tend to be associated with primary and secondary forest habitats, with a few species, including several tramp species (e.g., *Strumigenys emmae* Emery, 1890, *Strumigenys membranifera* Emery, 1869), relatively common in open and disturbed habitats such as urban parks (Kitahiro et al. 2014; Tang et al. 2019). At the microhabitat level, *Strumigenys* are typically encountered within the leaf-litter covering forests floors, though a few species nest under bark or epiphytes (Longino 2006), are associated with the accumulated leaf litter in trees (Nadkarni and Longino 1990) or forage on the understory vegetation (Lattke et al. 2018). In general, workers of *Strumigenys* species are collected by leaf litter extractions with Berlese funnels and mini-Winklers. However, these sampling methods may limit the discovery of some *Strumigenys* species, especially those with subterranean habits. The extraction of soil monoliths and the use of subterranean traps are effective methods to sample the poorly known subterranean ant fauna (Andersen and Brault 2010; Wong and Guénard 2017; Martins et al. 2020). An increase in their systematic use is likely to uncover new species, including within the genus *Strumigenys*.

Macao is a special administrative region of China located on the south side of the Pearl River Delta. Despite being an under-sampled and heavily urbanized territory with a land area of ~30 km^2^, it nevertheless harbors a surprisingly high ant diversity (Leong et al. 2017). Until recently, few species of *Strumigenys* were known to inhabit the region. The first *Strumigenys* record was made by Wheeler in 1928, with the mention of the exotic *Strumigenys membranifera*. Seventy-eight years later, a second record, *Strumigenys sylvestrii* Emery, 1906, was published by Hua (2006). This record is, however, most certainly erroneous (Tang et al. 2019). In 2017, opportunistic leaf litter extractions and hand collection expanded the list of *Strumigenys* species for Macao with four additional species records (Leong et al. 2017). Thus prior to this study, two native (i.e., *Strumigenys exilirhina* Bolton, 2000 and *Strumigenys minutula* Terayama & Kubota, 1989) and three introduced *Strumigenys* species (i.e., *S. emmae*, *S. membranifera*, and *S. nepalensis*) had been recorded in Macao, far less than the 24 species recorded in the neighboring territory of Hong Kong (Tang et al. 2019).

In this study, we used specimens collected through a holistic sampling protocol done to assess the ant fauna of Coloane Island, Macao (Brassard et al. unpublished). In particular, we focus on the *Strumigenys* species found within the region and report four new species records. Moreover, we describe a species collected with a new type of subterranean trap (M.K.L. Wong, unpublished): *Strumigenys subterranea* sp. nov. When available, we also provide new sociometric and ecological information for the species collected. Finally, we provide a taxonomic key for the 29 *Strumigenys* species known from the Guangdong-Hong Kong-Macao Greater Bay Area, a megacity including Macao SAR, Hong Kong SAR, and nine cities in Guangdong province (Hui et al. 2018).

## Materials and methods

The majority of specimens examined were collected in 2019 across multiple sites in Coloane Island, Macao (22.1261°N, 113.5669°E; Suppl. material 1: Fig. S1). Ants were sampled using a variety of methods including hand collection, arboreal traps, subterranean traps (Suppl. material 2: Fig. S2), leaf litter extraction with Winkler extractors, artificial ground nests (Booher et al. 2017) placed in the field between 11 and 18 weeks (Suppl. material 3: Fig. S3), and ground baits (Suppl. material 4: Fig. S4).

Images were taken with a Leica DFC450 camera mounted on a Leica M205 C dissecting microscope. Image montages of the specimens were taken, stacked, enhanced and measured using the Leica Application Suite v. 4.5.

## Results

### Taxonomic accounts

#### Strumigenys subterranea

Taxon classificationAnimaliaHymenopteraFormicidae

Brassard, Leong & Guénard sp. nov.

**Figure 1.**
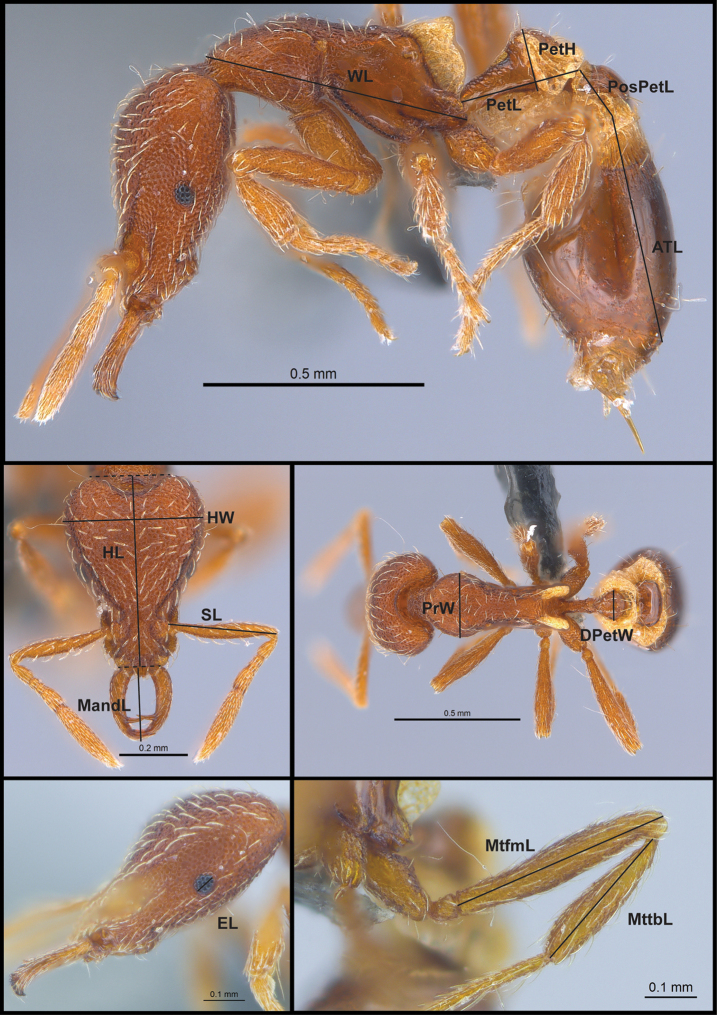
Morphological measurements used. For definition of each abbreviation see Table 1.

5FA0F7EB-49FE-5A94-933C-E711ED2356C3

http://zoobank.org/6229098D-6815-4ABB-9753-1D1B625FC215

##### Type locality.

Macao SAR, China: Coloane Island, Coloane North East hiking trail, 22.1351°N, 113.5700°E, ca. 80 m, subterranean trap placed at a depth of 12.5 cm, 14 May–4 June 2019, F. Brassard leg.

##### Repository institution.

Insect Biodiversity and Biogeography Lab (IBBL), School of Biological Sciences, Hong Kong University

##### Type specimen.

***Holotype*.** Pinned worker. Original label: “China SAR: Macau, Coloane. Coloane North East Hiking Trail. 78 m, 14v–4vi.2019, 22.13510°N, 113.57000°E, Subterranean Trap 12.5 cm depth. F. Brassard” “MAC_S12_12.5_q4_Sp.2” [IBBL: ANTWEB1010847].

##### Worker measurements.

(*n* = 1): TL 1.809 mm, HL 0.454 mm, HW 0.348 mm, CI 77, MandL 0.098 mm, MI 22, SL 0.226 mm, SI 65, PrW 0.189 mm, PI 54, EL 0.006 mm, OI 2, WL 0.458 mm, PetH 0.124 mm, PetL 0.183 mm, MtfmL 0.295 mm, MttbL 0.230 mm, LPI 68, DPetW 0.117 mm, DPI 64, PosPetL 0.184 mm, ATL 0.356 mm.

**Table 1.** Morphological measurements used. Morphological terminology follows Tang et al. (2019).

**TL**	**Total Length**: measured from the mandibular apex to the posterior margin of abdominal tergite IV. Sum of MandL + HL + ML + PetL + PosPetL + ATL.
**HL**	**Head Length**: measured from the midpoint of the occipital margin to the midpoint of the anterior clypeal margin. If one or both margins are concave, measured from the midpoint of a transverse line spanning the apices of the projecting portions.
**HW**	**Head Width**: measured at the maximum width of the head in full-face view, excluding the eyes.
**MandL**	**Mandible Length**: measured from the mandibular apex to the anterior clypeal margin. If clypeal margin concave medially, measured from the transverse line connecting the anteriormost points.
**SL**	**Scape Length**: measured from the basal constriction that occurs distal of the condylar bulb.
**EL**	**Eye Length**: maximum diameter of the eye.
**PrW**	**Pronotal Width**: maximum width of the pronotum in dorsal view. If present, projecting tubercles or other cuticular prominences at the pronotal humeral angles ignored.
**WL**	**Weber’s Length**: diagonal length of the mesosoma in profile view. Measured from the point at which the pronotum meets the cervical shield to the posterior basal angle of the metapleuron.
**PetL**	**Petiolar Length**: maximum length of petiole. Measured from posterior petiolar margin to the anteriormost point before posteroventral lobes of the propodeum obscure petiole. If present, spongiform tissues are ignored.
**PetH**	**Petiolar Height**: maximum distance measured between two parallel lines, one tangent with the node apex and the other tangent with the ventral-most point of the petiole in profile. If ventral margin concave upward, measure from the lower line tangent to the uppermost portion of the curve. If present, spongiform tissues ignored.
**DPetW**	**Dorsal Petiolar Width**: maximum width of petiolar node in dorsal view.
**PosPetL**	**Postpetiole Length**: maximum length of postpetiole, measured from the anterior margin to the posterior margin. If present, spongiform tissues are ignored.
**ATL**	**Abdominal tergum IV Length**: maximum length of the fourth abdominal tergite, measured from the anterior margin to the posterior margin.
**MtfmL**	**Metafemur length**: maximum length of the metafemur, not including the trochanter.
**MttbL**	**Metatibia length**: maximum length of the metatibia.
**CI**	**Cephalic Index**: HW / HL × 100
**MI**	**Mandibular Index**: MandL / HL × 100
**SI**	**Scape Index**: SL / HW × 100
**PI**	**Pronotum Index**: PrW/ HW x 100
**OI**	**Ocular Index**: EL / HW × 100
**LPI**	**Lateral Petiolar Index**: PetH / PetL × 100
**DPI**	**Dorsal Petiolar Index**: DPetW / PetL × 100

##### Diagnosis.

Mandibles in full-face view triangular, eyes with a single ommatidium, anterior margin of clypeus shallowly convex, clypeal margin fringed with a continuous row of appressed spatulate hairs incurved towards midline of head, conspicuous preocular carina, dorsoventrally flattened scape, spatulate to spoon-shaped hairs on leading edge of scape, pair humeral hairs present, dorsum of head behind clypeus reticulate-punctate, side of mesosoma and disc of postpetiole smooth, postpetiole with concave anterior margin and a projecting lobe on convex posterior margin, total dental count of eight, lack of propodeal spines, and propodeal declivity angular.

##### Worker description.

**Figure 2.**
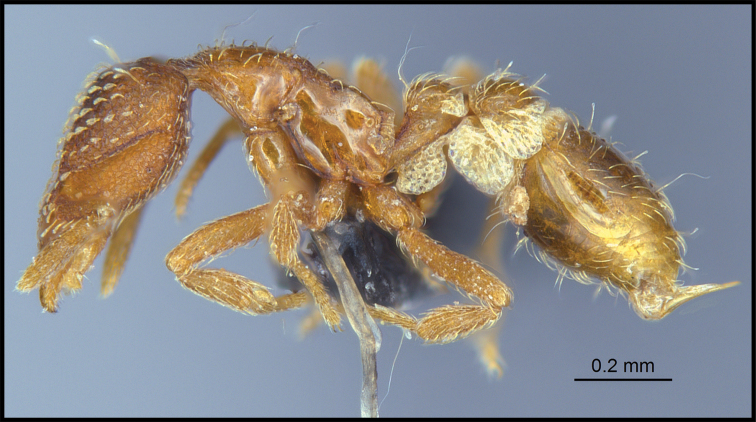
*Strumigenys subterranea* sp. nov. (ANTWEB1010847), worker in profile view.

**Figure 3.**
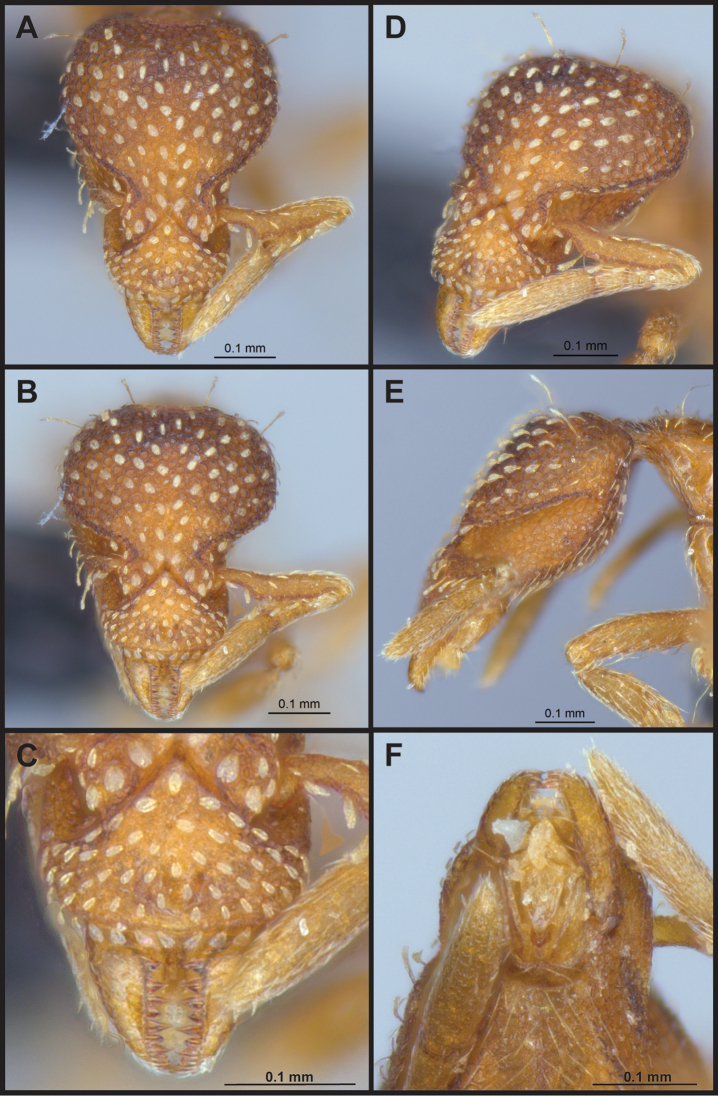
*Strumigenys subterranea* sp. nov. (ANTWEB1010847) **A–F** worker **A** full-face view **B** face view tilted posteriorly to showcase hairs on vertex **C** mandibles **D** left antenna **E** side view to showcase the eye **F** ventral view to showcase labrum.

**Figure 4.**
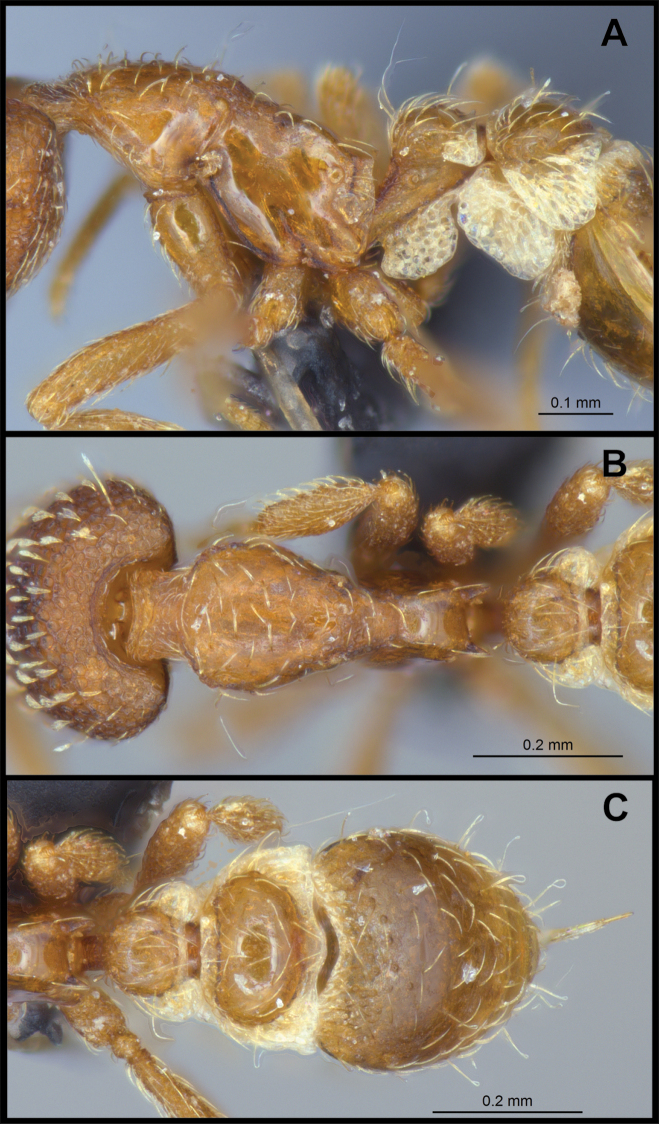
*Strumigenys subterranea* sp. nov. (ANTWEB1010847) **A–C** worker **A** lateral view of mesosoma **B** dorsal view of mesosoma **C** dorsal view of metasoma.

(Figs 2–4). ***Head.*** In full-face view, head noticeably longer than wide (CI: 77) (Fig. 3A), with its widest portion nearby the anterior end of the posterior third of its length. In lateral view, eye with a single facet, inconspicuous, and located at the widest level of the head (Fig. 3E). Posterior cephalic margin shallowly concave; corners of posterior margin of head weakly developed and evenly rounded through the lateral margins. Posterolateral margins evenly rounded on half of their length, then converging at a slightly steeper angle towards the center of the head. Anteromedian clypeal margin slightly convex. Scapes with a moderately developed subbasal lobe on their anterior portion. Apex of scape not reaching posterior margin of head, antenna including scape with six articles, with the last two articles distinctly enlarged and forming a club; ratio of antennal segments from 2^nd^ to 6^th^ segment = 3.83 : 1.33 : 1 : 3.05 : 11.56 (Fig. 3D). Mandibles triangular with eight teeth (T) and four denticles (D), arranged from basal to apical as such: T-D-T-T-T-T-T-T-D-D-D-T (Fig. 3C). Basal angle between the basal margin and masticatory margin rounded triangular. Basal lamella a thin strip, widest at the basal tooth and almost fully disappearing at the midpoint of the masticatory margin. Labrum terminates in a pair of short triangular lobes (Fig. 3F).

***Mesosoma*.** In lateral view, dorsum of mesosoma broadly convex but slightly concave at the metanotal groove (Fig. 4A). Anterior portion of promesonotum in dorsal view convex (Fig. 4B), with its widest point slightly posterior to the humeral hairs. Median anterior margin of promesonotum slightly convex. Lateral margin of premosonotum subparallel and slightly convex. Metanotal groove distinct but weakly incised. In dorsal view, propodeum approximately half of the maximal width of the promesonotum. In lateral view, propodeum with an angular declivity. Propodeal declivity with a spongiform lamella.

***Metasoma*.** Petiole in lateral view elongate (LPI: 68) and subclavate, with long and thin peduncle. Petiolar node well developed; dorsum of node convex, with its widest point at the posterodorsal corner. Petiolar node in dorsal view subcircular (DPI: 64), widest towards the posterior part. Postpetiolar disc in dorsal view suboval and distinctly wider than long; the median portion of the anterior margin distinctly concave whereas the posterior margin convex with a lobe projecting from the median portion (Fig. 4C). Limbus in dorsal view strongly concave, with a thin spongiform pad along its length. Spongiform tissues present on both petiole and postpetiole. Spongiform tissue on the lateral side of petiole restricted to the posterior portion of the node in profile. Excluding the anteriormost part of the ventral portion of petiole, spongiform tissue covers the ventral portion of both the petiole and postpetiole entirely. Depth of spongiform tissue under petiole nearly as much as petiole height. Spongiform tissue particularly extensive on the ventral lobes of the postpetiole. In dorsal view spongiform tissue present along the posterior margin of the petiolar node, and surrounding disc of postpetiole.

***Pilosity*.** On head, spatulate hairs arising from their base and then abruptly curving towards the mandibles, forming a space between the scale of the hair and the head surface. In full-face view of head, numerous evenly spaced spatulate hairs (ca. 95) along the frons, with around two-thirds as much spatulate hairs (ca. 60) evenly spaced but more densely arranged on the clypeus. A total of 16 smaller spatulate hairs present on anterior margin of clypeus. On each side of the anterior margin of the clypeus, three hairs on lateral portion and five on the anterior portion, all incurved towards the midline of the head. Largest spatulate hairs (*n* = 6) fully extending and found on subbasal lobe of antennal scape; with the first two basal hairs curved towards the apex of the scape, whereas the four most posterior hairs are curved towards the base of the scape. Two pairs of thin remiform hairs on the vertex; with one pair on the lateral portions of vertex and the other in posteromedial position (Fig. 3B). In profile view, appressed simple hairs present below antennal scrobe towards ventral portion of head. On the mesosoma and metasoma, decumbent hairs evenly spaced with a pair of long flagellate humeral hair present on petiolar node; several erected simple, appressed and filiform hairs present on first gastral tergite, whereas other tergites and sternites are mostly covered by appressed simple hairs. Appressed simple hairs present on tibia, femur and tarsus. Meso- and meta-basitarsal hairs flagellate. Flagellate hairs absent from femurs and tibias.

***Sculpture*.** In full-face and lateral view, head covered by areolate sculpturing (0.10 – 0.23 mm). In dorsal view, superficial sculpturing on the surrounding of the promesonotum and on its posterior section. Center of the dorsal portion of the promesonotum and propodeum smooth; lateral portions of mesosoma smooth (Fig. 4A). In dorsal view, discs of petiole and postpetiole smooth. In lateral view, petiole with weak sculpturing. Basigastral costulae present as weakly developed and irregular imprints on the central part of the limbus, extending around half the length of the postpetiole disc. Sculpturing on tibias and femurs areolate. Leg bullae absent.

***Color*.** Body coloration concolor yellowish brown, with slightly lighter coloration on the legs, antennae, mandibles and at the apex of the gaster. First gastral tergite and sternite with darker coloration.

##### Comments.

*Strumigenys subterranea* sp. nov. belongs to the *Strumigenys rostrata* group of the Malesian-Oriental-East Palearctic region (Bolton 2000), due to a combination of morphological characters: mandibles in full-face view triangular, basal lamella of mandible low and rounded-triangular, anterior margin of clypeus broad and shallowly convex, clypeal margin fringed with a continuous row of curved spatulate to spoon-shaped hairs, conspicuous preocular carina, dorsoventrally flattened scape, spatulate to spoon-shaped hairs on leading edge of scape, cuticle within scrobe reticulate or reticulate-punctate, pronotum dorsum without a median longitudinal carina, spongiform appendages present on petiole and postpetiole, pronotal humeral hair present, dorsum of head behind clypeus reticulate-punctate, side of propodeum and disc of postpetiole smooth.

However, *S. subterranea* sp. nov. can be distinguished from the other 17 species within this group (Table 2) by a combination of the following characters: eyes with a single ommatidia, clypeal margin shallowly convex, evenly spaced appressed spatulate hairs along frons (with appressed spatulate hairs evenly spaced but more densely arranged on clypeus), postpetiole with concave anterior margin and a projecting lobe on convex posterior margin, total dental count of nine, lack of propodeal spines, and propodeal declivity angular, not rounded.

**Table 2.** Comparison of five diagnostic characters for *S. rostrata* group. Characters are (A) appressed spatulate hairs on cephalic region, (B) margin of clypeus convex, (C) anterior margin of postpetiole concave, (D) propodeal spines small or absent and (E) eye composed of a single ommatidium. Values represent the presence (1) or absence (0) of a character.

**Species**	**Characters**				
	**A**	**B**	**C**	**D**	**E**
* subterranea *	1	1	1	1	1
* ambatrix *	1	1	1	0	0
* arizonica *	0	0	0	0	0
* atropos *	0	0	0	1	1
* Bunki *	1	0	0	1	0
* californica *	1	0	0	0	0
* carolinensis *	1	0	0	0	0
* chiricahua *	0	0	0	0	0
* emeswangi *	0	0	1	1	0
* Fautrix *	1	1	1	0	0
* hyalina *	1	0	0	0	0
* incerta *	0	0	1	1	0
* inopina *	0	0	1	0	0
* nepalensis *	1	0	1	0	0
* rostrata *	1	0	0	0	0
* rostrataeformis *	1	1	0	0	0
* symmetrix *	1	1	1	0	0
* Victrix *	1	0	1	1	0

We found that the most peculiar characteristic of *S. subterranea* (i.e., having very small eyes) is shared with *Strumigenys atropos* Bolton, 2000. However, the shape of the postpetiolar node (straight anterior margin in *S. atropos* but concave in *S. subterranea* sp. nov.) and the shape of the anterior margin of the clypeus differ (slightly convex in *S. subterranea* sp. nov., but noticeably concave in *S. atropos*). Moreover, large spatulate hairs are present up to two-third of the length of the lateral margins of the head of *S. atropos*, but not in *S. subterranea.* Lastly, large spatulate hairs are present on the dorsal portion of the pronotum of *S. atropos*, whereas hairs on the dorsal portion of *S. subterranea* are fine.

##### Etymology.

The name of this new species refers to the stratum it was collected in and to its suggested subterranean ecology.

##### Ecology.

A single worker from this species has been collected so far, found within a subterranean trap; a 15 mL falcon tube placed at a depth of 12.5 cm below the ground surface. It contained ethanol 70% and was baited with tuna mixed with honey (see Suppl. material 1: Fig. S1 for sampling design). The trap was placed in young secondary forest and was operating continuously for a period of 21 days. Little is known about the ecology of this species. However, due to the extremely reduced eyes present on the specimen and its collection through a subterranean trap, it is here suggested that the species has subterranean habits. Further reinforcing this hypothesis is the fact that extensive sampling in Hong Kong and Macao over the past 6 years focusing on ground-dwelling and leaf-litter ants using Winklers and pitfall traps never yielded this species. Nevertheless, only a single worker was found within one out of 256 subterranean traps retrieved during our sampling on Coloane Island, which indicates this species is uncommon. Our data also suggests it cohabits within the same soil layer with other ant species, including other subterranean species. Indeed, we found within the same trap one worker of *Pheidole ochracea* Eguchi, 2008 and hundreds of workers of *Carebara zengchengensis* Zhou, Zhao & Jia, 2006. Additionally, within the same quadrat (1 × 1 m) we also found *C. zengchengensis* at depths of 25, 37.5 and 50 cm, as well as *Solenopsis jacoti* Wheeler, 1923 and *Buniapone amblyops* Emery, 1887 at a depth of 50 cm.

#### Strumigenys elegantula

Taxon classificationAnimaliaHymenopteraFormicidae

Terayama & Kubota, 1989

F7790D46-F1E7-5DC1-91CC-BFD84E6E4856

Figure 5

Smithistruma elegantula Terayama & Kubota, 1989: 788, figs 23–27 (w.q.) TAIWAN. Indomalaya
Pyramica elegantula (Terayama & Kubota, 1989). Combination in Pyramica: Bolton 1999: 1673.
Strumigenys elegantula (Terayama & Kubota, 1989). Combination in Strumigenys: Baroni Urbani and De Andrade 2007: 119.

##### Geographic distribution.

China (Guangdong, Guangxi, Hong Kong, Macao, Taiwan), Thailand.

**Figure 5.**
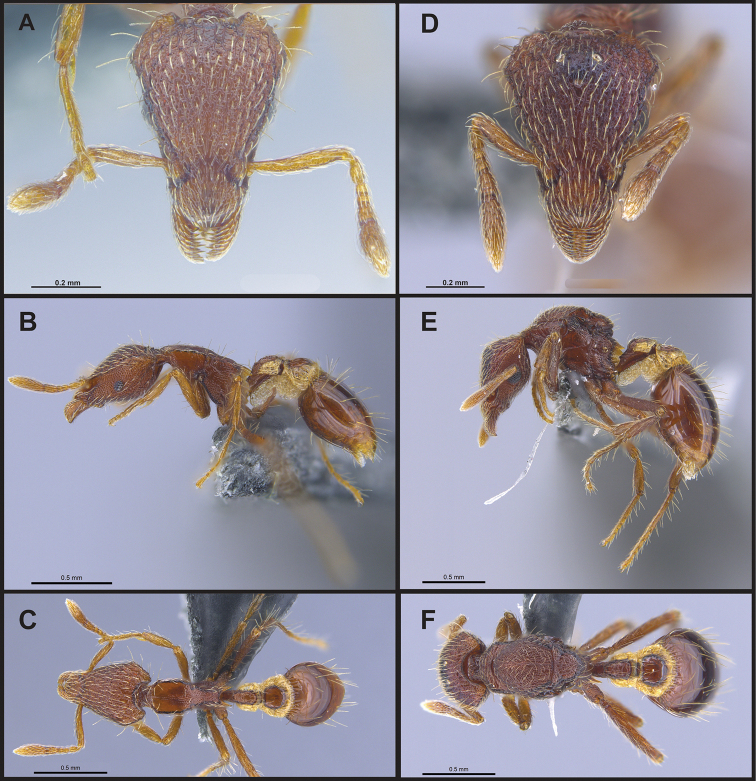
*Strumigenys elegantula* in full-face, profile and dorsal view **A–C** worker (MAC_S04_LLSP_sp.9) **D–F** queen (MAC_S04_LLSP_sp.9).

##### Comments.

This is a new species record for Macao. Originally described from Taiwan, this species is more widespread within continental Asia since it has also been recorded in Hong Kong, Macao, Guangdong, Guangxi (China) as well as in Thailand. In both Macao and Hong Kong (Tang et al. 2019), this species is relatively common and is known from two and ten sites within these regions, respectively.

##### Material examined.

Macao SAR, China • 28 Workers; Macao, Coloane Island, Ka Ho; 22.1294°N, 113.5914°E, ca. 30 m; 20 Mar. 2019; F. Brassard leg.; Winkler; MAC_S04_LLSP_Sp.9; IBBL. • 1 Worker; Macao, Coloane Island, Ka Ho; 22.1294°N, 113.5914°E, ca. 30 m; 20 Mar. 2019; F. Brassard leg.; Winkler; MAC_S04_LLSA_Sp.1; IBBL. • 1 Worker; Macao, Coloane Island, Ka Ho Family Trail Peak; 22.1284°N, 113.5702°E, ca. 180 m; 16 May 2019; F. Brassard leg.; Winkler; MAC_S14_LLSP_Sp.1; IBBL. • 1 Worker; Macao, Coloane Island, Ka Ho Family Trail Peak; 22.1284°N, 113.5702°E, ca. 180 m; 16 May 2019; F. Brassard leg.; Winkler; MAC_S14_LLSA_Sp.3; IBBL. • 1 Worker; Macao, Coloane Island, Ka Ho Height Family trail peak near 1-09-03; 22.1284°N, 113.5702°E, ca. 140 m; 16 May 2019; F. Brassard leg.; Ground Bait; MAC_S14_B06_Sp.1; IBBL. • 1 Queen; Macao, Coloane Island, Ka Ho; 22.1294°N, 113.5914°E, ca. 30 m; 20 Mar. 2019; F. Brassard leg.; Winkler; MAC_S04_LLSP_Sp.9; IBBL.

#### Strumigenys emmae

Taxon classificationAnimaliaHymenopteraFormicidae

Emery, 1890

6DD91D3A-8160-57A4-9488-D2EBC688F047

Figure 6

Epitritus emmae Emery, 1890: 70, pl. 8, fig. 6 (w.) Antilles. Neotropics.
Quadristruma emmae (Emery, 1890). Combination in Quadristruma: Brown 1949: 48.
Strumigenys emmae (Emery, 1890). Combination in Strumigenys: Bolton 1999: 1674.

##### Geographic distribution.

*Native*: Australia. *Introduced*: Widespread, Afrotropical, Malagasy, Nearctic, Neotropical, Oceanian, Oriental, Panamanian, Saharo-Arabian realms, see antmaps.org for a global account (Janicki et al. 2016; Guénard et al. 2017). Within China, found in Hong Kong and Macao.

**Figure 6.**
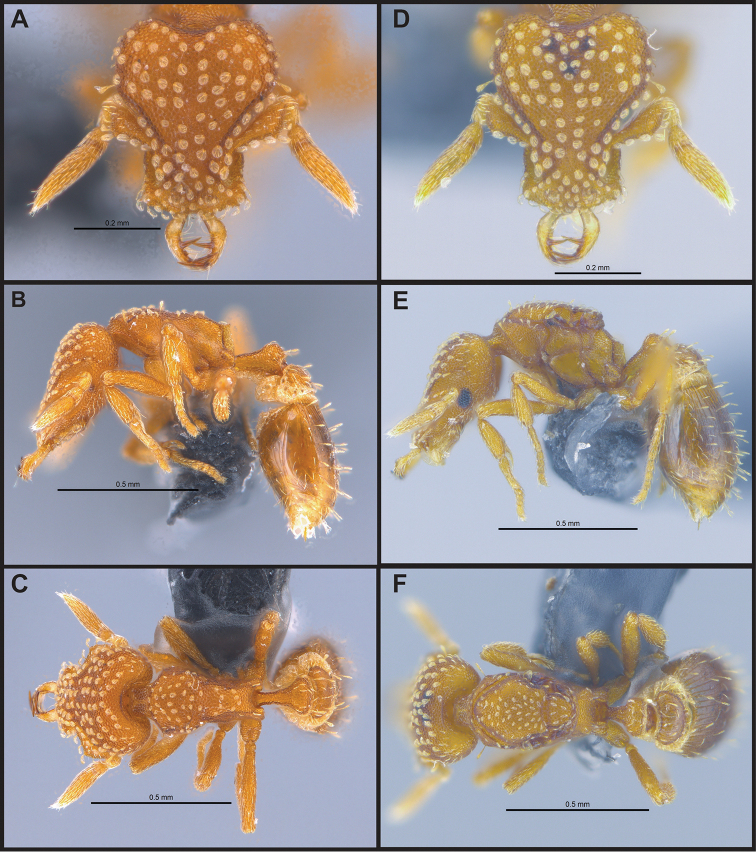
*Strumigenys emmae* in full-face, profile and dorsal view **A–C** worker (MAC_S20_LLSP_Sp.7) **D–F** queen (MAC_S19_LLSA_Sp.1).

##### Comments.

Originally from Australia, *S. emmae* is now a widespread exotic species. Although the exact date at which this species was introduced in the region is unknown, it is known from Hong Kong since the 1990s (Fellowes 1999), and was more recently recorded from Macao (Leong et al. 2017).

##### Material examined.

Macao SAR, China • 3 Workers; Macao, Coloane Island, Caesars Golf Macau, 22.1351°N, 113.5611°E, ca. 10 m, 25 June 2019, MAC_S19_LLSA_Sp.1, F. Brassard leg., Winkler; IBBL. • 2 Workers; Macao, Coloane Island, Cotai Ecological Zone II; 22.1418°N, 113.5519°E, ca. 0 m; 26 June 2019; F. Brassard leg.; Winkler; MAC_S20_LLSA_Sp.6; IBBL • 3 Workers; Macao, Coloane Island, Cotai Ecological Zone II; 22.1418°N, 113.5519°E, ca. 0 m; 26 June 2019; F. Brassard leg.; Winkler; MAC_S20_LLSP_Sp.7; IBBL. • 1 Worker; Macao, Taipa Island, Siu Tam Hill; 22.1608°N, 113.5466°E, ca. 80 m; 15 Aug. 2018; C.M. Leong leg.; CML-FW-15viii2018; IBBL. • 1 Worker; Macao, Macao Peninsula, Guia Hill; 22.1983°N, 113.5511°E, ca. 60 m; 18 Aug. 2018; C.M. Leong leg.; IBBL. • 1 Queen; Macao, Coloane Island, Caesars Golf Macau; 22.1351°N, 113.5611°E, ca. 10 m; 25 June 2019; F. Brassard leg.; Winkler; MAC_S19_LLSA_Sp.1; IBBL]. • 1 Queen; Macao, Taipa Island, Siu Tam Hill; 22.1608°N, 113.5466°E, ca. 80 m; 26 Aug. 2016; C.M. Leong leg.; IBBL.

#### Strumigenys exilirhina

Taxon classificationAnimaliaHymenopteraFormicidae

Bolton, 2000

87A8A597-A277-5010-98B1-C6D7EC155096

Figure 7

Strumigenys exilirhina Bolton, 2000: 881 (w.q.) Nepal. Indomalaya.

##### Geographic distribution.

**Figure 7.**
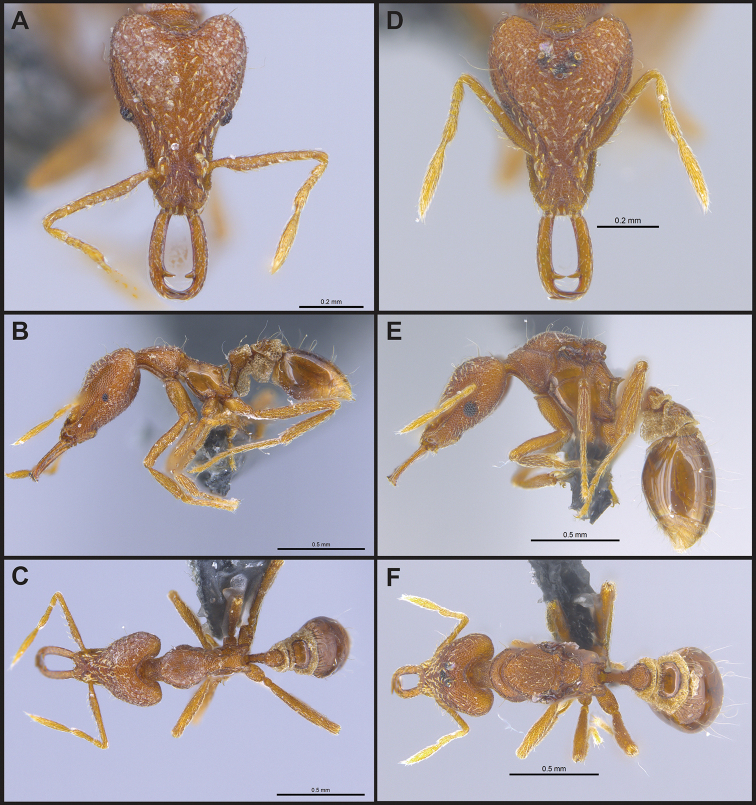
*Strumigenys exilirhina* in full-face, profile and dorsal view **A–C** worker (MAC_S01_LLSA_Sp.3) **D–F** queen (MAC_S16_LLSA_Sp.9).

Bhutan, China (Guangdong, Hong Kong, Jiangxi, Macao, Xizang, Yunnan), India, Japan, Nepal, Thailand.

##### Comments.

This species, first recorded in Macao in 2017 (Leong et al. 2017), is one of the most commonly collected *Strumigenys*. During the 2019 survey, it was found at 12 different sites within nature parks. In Hong Kong, it is recorded from various habitats including disturbed urban forests, tree plantations, shrubland, secondary forests and Feng Shui woods (Tang et al. 2019).

##### Material examined.

Macao SAR, China • 3 Workers; Macao, Coloane Island, Coloane Park; 22.1214°N 113.5649°E, ca. 110 m; 18 Mar. 2019; F. Brassard leg.; Winkler; MAC_S01_LLSA_Sp.3; IBBL • 2 Workers; Macao, Coloane Island, Hillside of Department of Green Areas and Gardens; 22.1275°N, 113.5612°E, ca. 70 m; 20 May 2019; F. Brassard leg.; Winkler; MAC_S16_LLSA_Sp.9; IBBL. • 1 Worker; Macao, Coloane Island, Wetland Alto de Coloane; 22.1230°N, 113.5597°E, ca. 90 m; 19 Apr. 2019; F. Brassard leg.; Winkler; MAC_S02_LLSA_Sp.7; IBBL. • 1 Worker; Macao, Coloane Island, Seoc Pai Van Park; 22.1249°N, 113.5566°E, ca. 40 m; 20 Mar. 2019; F. Brassard leg.; Winkler; MAC_S05_LLSP_Sp.7; IBBL. • 4 Workers; Macao, Coloane Island, Hac Sa Reservoir Family trail near 1-05-12; 22.1237°N, 113.5684°E, ca. 90 m; 8 Apr. 2019; F. Brassard leg.; Winkler; MAC_S06_LLSA_Sp.3; IBBL. • 7 Workers; Macao, Coloane Island, Hac Sa Reservoir Family trail near 1-05-12; 22.1237°N, 113.5684°E, ca. 90 m; 8 Apr. 2019; F. Brassard leg.; Winkler; MAC_S06_LLSP_Sp.4; IBBL. • 11 Workers; Macao, Coloane Island, Coloane trail near 1-01-10; 22.1165°N, 113.5589°E, ca. 100 m; 10 Apr. 2019; F. Brassard leg.; Winkler; MAC_S10_LLSA_Sp.4; IBBL. • 1 Worker; Macao, Coloane Island, Coloane trail near 1-01-15; 22.1151°N, 113.5645°E, ca. 80 m; 11 Apr. 2019; F. Brassard leg.; Winkler; MAC_S11_LLSA_Sp.4; IBBL. • 2 Workers; Macao, Coloane Island, Ka Ho height family trail peak near 1-09-03; 22.1284°N, 113.5702°E, ca. 140 m; 16 May 2019; F. Brassard leg.; Winkler; MAC_S14_LLSP_Sp.3; IBBL. • 2 Workers; Macao, Coloane Island, Oscar farm hillside; 22.1131°N, 113.5557°E, ca. 80 m; 24 June 2019; F. Brassard leg.; Winkler; MAC_S18_LLSA_Sp.11; IBBL. • 1 Worker; Macao, Macao Peninsula, Mongha Hill; 22.2085°N, 113.5476°E; 18 Feb. 2018; C.M. Leong leg.; CML-FW-18ii2018. • 1 Worker; Macao, Coloane Island, Ka Ho Reservoir; 22.1341°N, 113.5786°E; 27 Feb. 2018; C.M. Leong leg.; Winkler; CML-FW-27ii2018; IBBL. • 2 Workers; Macao, Coloane Island, Ka Ho Reservoir; 14 Aug. 2018; C.M. Leong leg.; IBBL]. • 1 Worker; Macao, Coloane Island, Hac Sa Reservoir; 22.1264°N, 113.5733°E; C.M. Leong leg.; IBBL. • 1 Queen; Macao, Coloane Island, Ka Ho Reservoir; 22.1608°N, 113.5466°E; 15 Jul. 2018; C.M. Leong leg.; Winkler; CML-FW-15vii2018; IBBL. • 1 Queen; Macao, Coloane Island, Hillside of Department of Green Areas and Gardens; 22.1275°N, 113.5612°E, ca. 70 m; 20 May 2019; F. Brassard leg.; Winkler; MAC_S06_LLSA_Sp.3; IBBL. • 2 Queens; Macao, Coloane Island, Coloane trail near 1-01-10; 22.1165°N, 113.5589°E, ca. 100 m; 10 Apr. 2019; F. Brassard leg.; Winkler; MAC_S10_LLSP_Sp.2; IBBL. • 1 Queen; Macao, Coloane Island, Hillside of Department of Green Areas and Gardens; 22.1275°N, 113.5612°E, ca. 70 m; 20 May 2019; F. Brassard leg.; Winkler; MAC_S16_LLSA_Sp.9; IBBL.

#### Strumigenys feae

Taxon classificationAnimaliaHymenopteraFormicidae

Emery, 1895

6C8A06A5-2CFF-5EAD-9783-318434DB81AA

Figure 8

Strumigenys feae Emery, 1895: 473 (w.q.) Myanmar. Indomalaya.

##### Geographic range.

Cambodia, China (Hong Kong, Macao, Yunnan), Myanmar, Thailand, and Vietnam.

**Figure 8.**
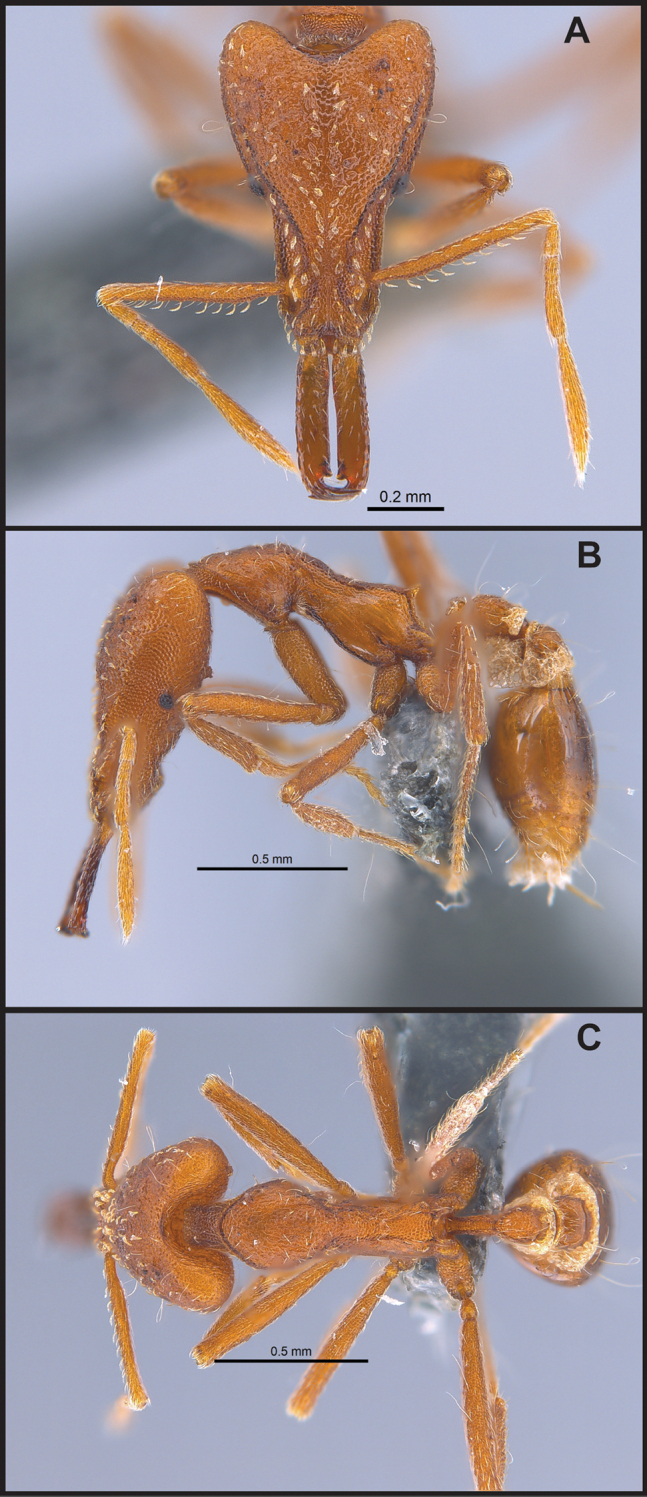
*Strumigenys feae***A–C** worker (MAC_S15_LLSP_Sp.8) **A** full-face view **B** profile view **C** dorsal view.

##### Comments.

A single worker of *S. feae* has been collected in Macao in 2019 (within a nature park consisting of young secondary forest), and as such the species is considered relatively rare in the region. In Hong Kong, *S. feae* has been collected in tree plantations of *Lophostemon confertus* Wilson & Waterh, 1982 and in secondary forests (Tang et al. 2019).

##### Material examined.

Macao SAR, China • 1 Worker; Macao, Coloane Island, Coastal Trail; 22.1144°N, 113.5699°E, ca. 110 m; 17 May 2019; F. Brassard leg.; Winkler; MAC_S15_LLSP_Sp.8; IBBL.

#### Strumigenys membranifera

Taxon classificationAnimaliaHymenopteraFormicidae

Emery, 1869

BA020175-EBF2-5C87-AF8F-7AD5D51FF51A

Figure 9

Strumigenys (Trichoscapa) membranifera Emery, 1869: 24, fig. 11 (w.) Italy. Palearctic.
Strumigenys (Cephaloxys) membranifera (Emery, 1869). Combination in Strumigenys (Cephaloxys): Emery 1916: 205.
Trichoscapa membranifera (Emery, 1869). Combination in Trichoscapa: Brown 1948: 113.
Pyramica membranifera (Emery, 1869). Combination in Pyramica: Bolton 1999: 1673.
Strumigenys membranifera (Emery, 1869). Combination in Strumigenys : Baroni Urbani and De Andrade 2007: 123.
Senior synonym of S. foochowensis, S. membranifera marioni, S. membranifera santschii, S. silvestriana, S. membranifera simillima, S. vitiensis, S. membranifera williamsi: Brown, 1948: 114.

##### Geographic distribution.

*Native*: Ghana, Sierra Leone, South Africa. *Introduced* : Widespread, Australasia, European, Indo-Malayan, Malagasy, Nearctic, Neotropical, Oceanian, Saharo-Arabian realms, see antmaps.org for a global account (Janicki et al. 2016). Within China, found in Guangdong, Hong Kong, Fujian, Macao, Sichuan, and Yunnan.

**Figure 9.**
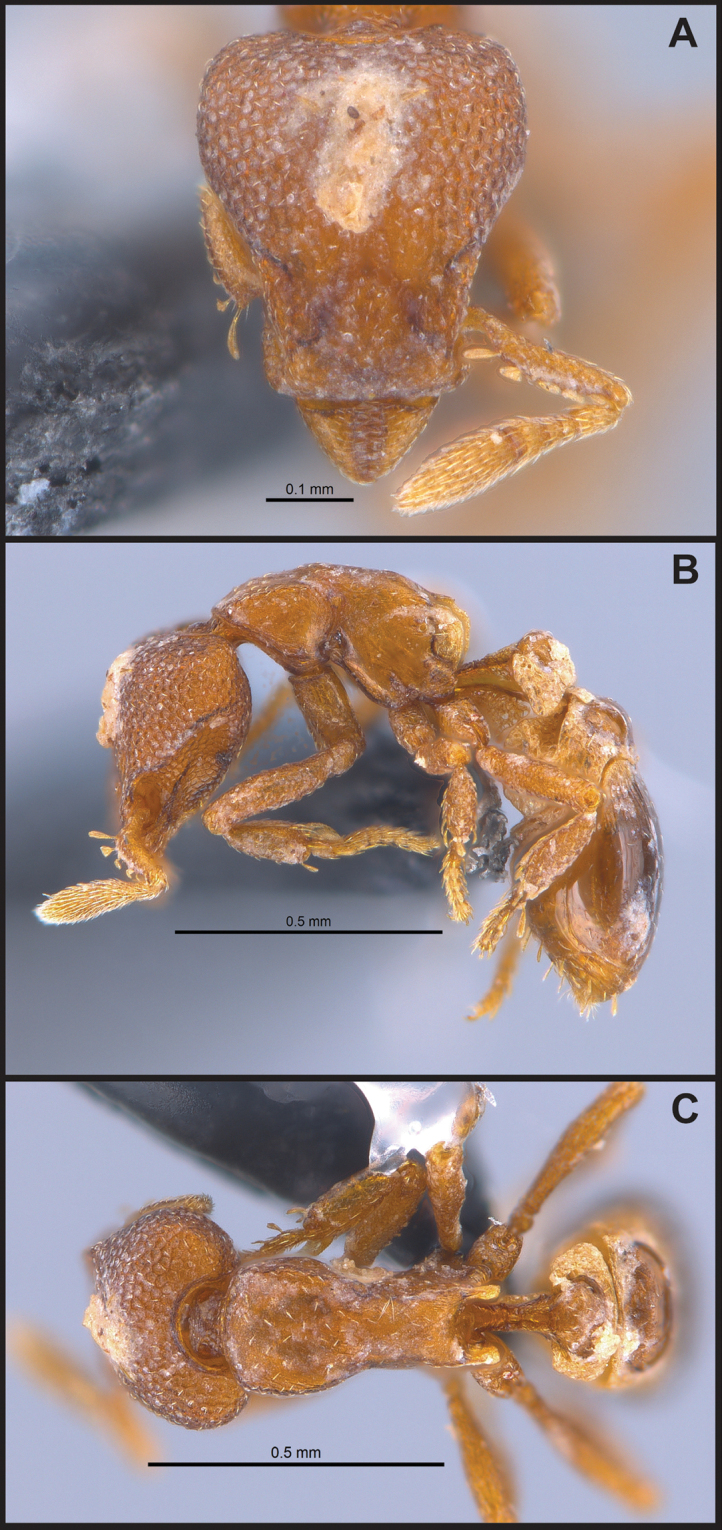
*Strumigenys membranifera***A–C** worker **A** full-face view **B** profile view **C** dorsal view.

##### Comments.

This species, originally from Africa, is associated with disturbed habitats. For instance, in Hong Kong it was collected near Disneyland and the Hong Kong Airport; two heavily disturbed localities (Tang et al. 2019). The exact date of its introduction in the region is unknown. It was first recorded in Hong Kong in 2019 (Tang et al. 2019), but has been known in Macao since 1928 where it was described from six specimens as *S. silvestriana* (Wheeler, 1928). As such, its initial introduction to the Greater Bay Area may date beyond a hundred years. Nevertheless, during the 2019 survey in Coloane the species was not frequently collected and was found only in two nature parks, which consisted of relatively small patches of young secondary forests within an urban matrix.

##### Material examined.

Macao SAR, China • 1 Worker; Macao, Coloane Island, Coloane Trail (Near C3 information point); 22.1217°N, 113.5560°E, ca. 110 m; 27 June 2019; F. Brassard leg.; Winkler; MAC_S21_LLSP_Sp.2; IBBL. • 2 Workers; Macao, Coloane Island, Morro de Hac Sa family trail near 1-07-08; 22.1144°N, 113.5699°E, ca. 50 m; 5 June-11 Sep 2019; F. Brassard leg.; Ground Nest; MAC_S15_GN3_H3_n1; IBBL.

#### Strumigenys minutula

Taxon classificationAnimaliaHymenopteraFormicidae

Terayama & Kubota, 1989

B2A567CD-48E0-5075-AA81-04023850B972

Figures 10 , 11

Strumigenys minutula Terayama & Kubota, 1989: 782, figs 13–17 (w.q.) Taiwan. Indomalaya.

##### Geographic distribution.

China (Hong Kong, Macao, Taiwan), Japan (Ryukyu Islands).

##### Comments.

**Figure 10.**
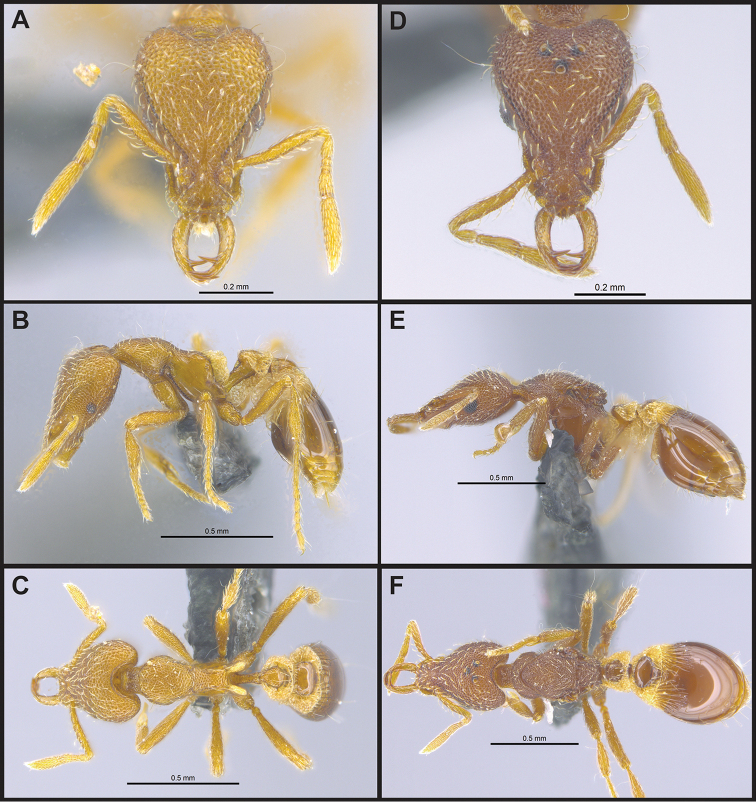
*Strumigenys minutula* in full-face, profile and dorsal view **A–C** worker (MAC_S11_GN3_H4_n1) **D–F** queen (MAC_S11_GN3_H4_n1).

**Figure 11.**
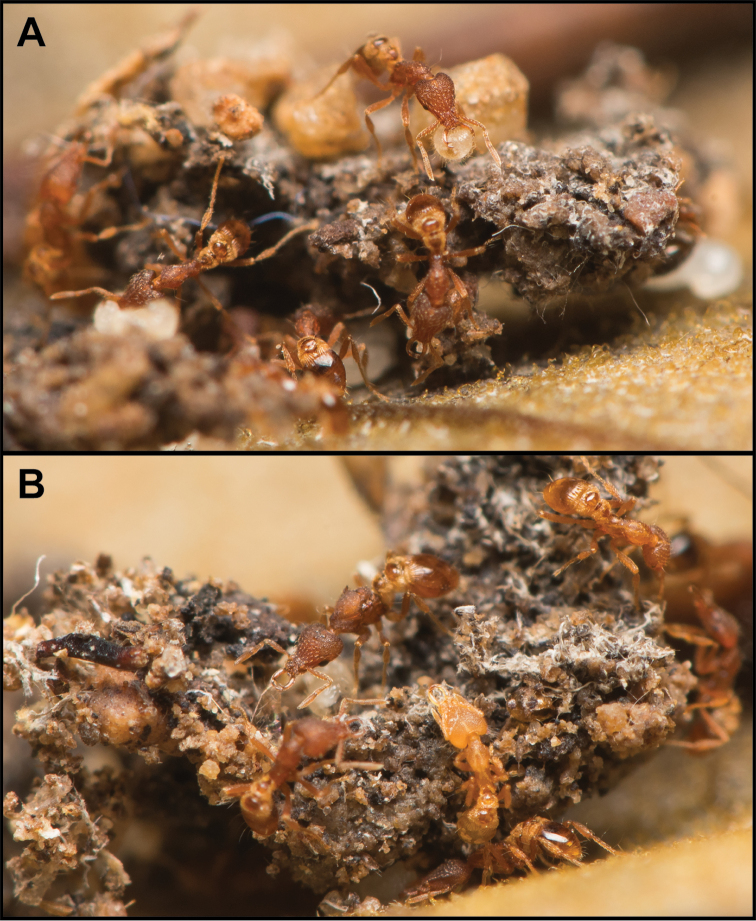
Photos of *S. minutula* (MAC_S11_GN3_H4_n1) showing some of the workers, including one carrying a larva (**A**), a queen (see center of **B**), and debris (**A–B**) found inside a ground nest.

In contrast to Hong Kong, where this species has been rarely collected (Tang et al. 2019), *S. minutula* was frequently found in Macao since its first collection in 2017 (Leong et al. 2017). Individuals were commonly found within leaf litter samples, and a full colony was also retrieved within one of the ground nests deployed (see Fig. 11). At the time of collection on (29 August 2019), the colony consisted of 135 workers, two dealate queens, eight larvae, and 12 pupae (eggs were not counted). This represents a similar colony size as described previously from Japan, where a polygynous colony of 300 individuals was recorded (Terayama et al. 2014). Note that, on the left foreleg of the queen imaged (Fig. 10E), a mite is attached. It remains to be investigated which mite species it is, and if this represent a case of parasitism, phoresis or myrmecophily in *S. minutula*.

##### Material examined.

Macao SAR, China • 22 Workers; Macao, Coloane Island, Hac Sa Reservoir family trail near 1-05-12; 22.1237°N, 113.5684°E, ca. 90 m; 8 April 2019; F. Brassard leg.; Winkler; MAC_S06_LLSA_Sp.6; IBBL. • 1 Worker; Macao, Coloane Island, Coloane trail near 1-01-10; 22.1165°N, 113.5589°E, ca. 100 m; 10 April 2019; F. Brassard leg.; Winkler MAC_S10_LLSA_Sp.2; IBBL. • 2 Workers; Macao, Coloane Island, Ka Ho Family Trail Peak; 22.1284°N, 113.5702°E, ca. 180 m; 16 May 2019; F. Brassard leg.; Winkler; MAC_S14_LLSP_Sp.4; IBBL. • 135 Workers; Macao, Coloane Island, Coloane Trail (Near 1-01-10 distance post); 22.1351°N, 113.5700°E, ca. 80 m; 16 May 2019; F. Brassard leg.; Ground nest; MAC_S11_GN3_H4_n1; IBBL. • 1 Worker; Macao, Coloane Island, Ka Ho Family Trail Peak; 22.1284°N, 113.5702°E, ca. 180 m; 16 May 2019; F. Brassard leg.; Winkler; MAC_S14_LLSA_Sp.11; IBBL]. • 1 Worker; Macao, Coloane Island, Ka Ho Lighthouse 2; 22.1292°N, 113.5909°E, ca. 30 m; 21 May 2019; F. Brassard leg.; Winkler; MAC_S17_LLSA_Sp.10; IBBL. • 13 Workers; Macao, Coloane Island, Ka Ho Lighthouse 2; 22.1292°N, 113.5909°E, ca. 30 m; 21 May 2019; F. Brassard leg.; Winkler; MAC_S17_LLSP_Sp.4; IBBL. • 1 Worker; Macao, Coloane Island, Caesars Golf Macau; 22.1351°N, 113.5612°E, ca. 10 m; 25 June 2019; F. Brassard leg.; Winkler; MAC_S19_LLSP_Sp.4; IBBL. • 1 Worker; Macao, Taipa Island, Siu Tam Hill; 22.1603°N, 113.5471°E; 22 July 2018; C.M. Leong leg.; IBBL. • 1 Worker; Macao, Coloane Island, Ka Ho Reservoir; 22.1251°N,, 113.5692°E; 20 July 2016; C.M. Leong leg.; IBBL. • 1 Worker; Macao, Coloane Island, Ka Ho Reservoir; 22.1251°N,, 113.5691°E; 20 August 2016; C.M. Leong leg.; IBBL. • 1 Worker; Macao, Coloane Island, Hac Sa Reservoir; 20 August 2016; C.M. Leong leg.; IBBL. • 2 Queens; Macao, Coloane Island, Coloane Trail (Near 1-01-10 distance post); 22.1351°N, 113.5700°E, ca. 80 m; 16 May 2019; F. Brassard leg.; MAC_S11_GN3_H4_n1; Ground nest; IBBL.

#### Strumigenys nepalensis

Taxon classificationAnimaliaHymenopteraFormicidae

Baroni Urbani & De Andrade, 1994

5856534C-9D31-52C2-9B38-1DEB008E8F6F

Figure 12

Strumigenys nepalensis Baroni Urbani & De Andrade, 1994: 57, figs 33, 34 (w.q.) Nepal. Indomalaya.
Smithistruma nepalensis (Baroni Urbani & De Andrade, 1994). Combination in Smithistruma: Bolton 1995: 385.
Pyramica nepalensis (Baroni Urbani & De Andrade, 1994). Combination in Pyramica: Bolton 1999: 1673.
Strumigenys nepalensis (Baroni Urbani & De Andrade, 1994). Combination in Strumigenys: Baroni Urbani and De Andrade 2007: 124.

##### Geographic distribution.

*Native*: China (Yunnan), India (north), Malaysia, Nepal, Singapore, Thailand, Vietnam. *Introduced*: China (Hong Kong, Macao), Mascarene Islands, India (Kerala).

**Figure 12.**
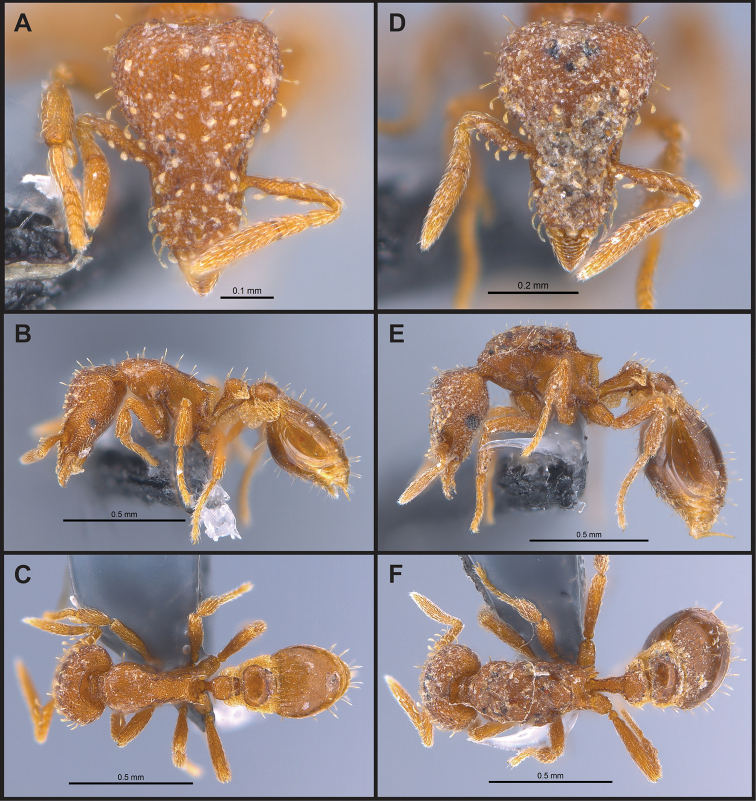
*Strumigenys nepalensis* in full-face, profile and dorsal view **A–C** worker (MAC_S19_LLSP_Sp.3) **D–F** queen (MAC_S19_LLSP_Sp.3).

##### Comments.

This species was first recorded in Macao in 2017 (Leong et al. 2017). In 2019, of the three sites at which *S. nepalensis* was collected from, two were heavily disturbed; they consisted of a golf course, and a thin strip of forest bordered by the ocean and a highway. The last one was a patch of young secondary forest nearby another golf course. Our records in Macao support the hypothesis of Tang and collaborators (2019) that this species is a potential tramp species adapted to human-disturbed habitats.

##### Material examined.

Macao SAR, China • 3 Workers; Macao, Coloane Island, Caesars Golf Macau; 22.1351°N, 113.5611°E, ca. 10 m; 25 June 2019; F. Brassard leg.; Winkler; MAC_S19_LLSP_Sp.3; IBBL. • 3 Worker; Macao, Coloane Island, Ka Ho Reservoir hillside; 22.1333°N, 113.5744°E, ca. 90 m; 9 April 2019; F. Brassard leg.; Winkler MAC_S09_LLSA_Sp.5; IBBL. • 21 Workers; Macao, Coloane Island, Caesars Golf Macau; 22.1351°N, 113.5612°E, ca. 10 m; 25 June 2019; F. Brassard leg.; Winkler; MAC_S19_LLSA_Sp.2; IBBL. • 19 Workers; Macao, Coloane Island, Cotai Ecological Zone II; 22.1418°N, 113.5519°E, ca. 0 m; 26 June 2019;F. Brassard leg.; MAC_S20_LLSA_Sp.7; Winkler; IBBL. • 1 Worker; Macao, Coloane Island, Cotai Ecological Zone II; 22.1418°N, 113.5519°E; ca. 0 m; 26 June 2019; F. Brassard leg.; Winkler; MAC_S20_LLSP_Sp.8; IBBL. Worker, Macao, Hac Sa Reservoir, 20 August 2016, C.M. Leong leg., [IBBL], (*n* = 1). • 4 Queens; Macao, Coloane Island, Caesars Golf Macau; 22.1351°N, 113.5612°E, ca. 10 m; 25 June 2019; F. Brassard leg.; Winkler MAC_S19_LLSA_Sp.2; IBBL. • 3 Queens; Macao, Coloane Island, Caesars Golf Macau; 22.1351°N, 113.5611°E, ca. 10 m; 25 June 2019; F. Brassard leg.; Winkler; MAC_S19_LLSP_Sp.3; IBBL. • 3 Queens; Macao, Coloane Island, Cotai Ecological Zone II; 22.1418°N, 113.5519°E, ca. 0 m; 26 June 2019; F. Brassard leg.; Winkler; MAC_S20_LLSA_Sp.7; IBBL. • 3 Queens; Macao, Coloane Island, Cotai Ecological Zone II; 22.1418°N, 113.5519°E, ca. 0 m; 26 June 2019; F. Brassard leg.; Winkler; MAC_S20_LLSP_Sp.8; IBBL.

#### Strumigenys sauteri

Taxon classificationAnimaliaHymenopteraFormicidae

Forel, 1912

F5E4E8D6-BF1E-5B56-B346-B6B88AD39659

Figure 13

Pentastruma sauteri Forel, 1912: 51 (w.) Taiwan. Indomalaya.
Pyramica sauteri (Forel, 1912). Combination in Pyramica: Bolton 1999: 1673.
Strumigenys sauteri (Forel, 1912). Combination in Strumigenys: Baroni Urbani and De Andrade 2007: 127.

##### Geographic distribution.

China (Fujian, Guangxi, Hong Kong, Hunan, Macao, Taiwan, Yunnan), Japan (Ryukyu Islands), Thailand.

**Figure 13.**
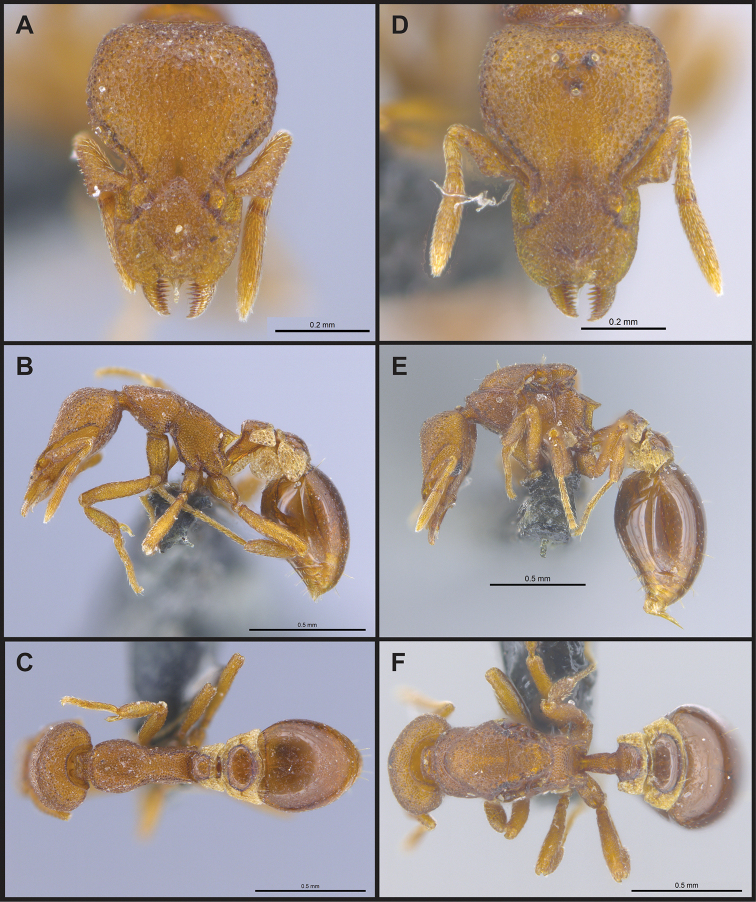
*Strumigenys sauteri* in full-face, profile and dorsal view **A–C** worker (MAC_S04_LLSP_sp.2) **D–F** queen (MAC_S11_LLSP_Sp.4).

##### Comments.

Although widely distributed in Hong Kong across multiple habitats, including shrublands, plantations, urban forest remnants, secondary forest and Feng Shui woods (Tang et al. 2019), this species appears less common in Macao where it has been collected at four different sites, all of which were relatively small patches of young secondary forests. This is a new species record for Macao.

##### Material examined.

Macao SAR, China • 1 Worker; Macao, Coloane Island, Ka Ho; 22.1936°N, 113.5914°E, ca. 30 m; 20 March 2019; F. Brassard leg.; Winkler; MAC_S04_LLSA_Sp.2; IBBL. • 5 Workers; Macao, Coloane Island, Coloane trail near 1-01-15; 22.1151°N, 113.5645°E, ca. 80 m; 11 April 2019; F. Brassard leg.; Winkler; MAC_S11_LLSP_Sp.4; IBBL. • 3 Workers; Macao, Coloane Island, Oscar Farm hillside; 22.1131°N, 113.5557°E, ca. 80 m; 24 June 2019; F. Brassard leg.; Winkler; MAC_S18_LLSA_Sp.10; IBBL. • 1 Worker; Macao, Taipa Island, Tai Tam Hill; 22.1578°N, 113.5679°E; 26 July 2018; C.M. Leong leg.; IBBL. • 1 Queen; Macao, Coloane Island, Coloane Trail (Near 1-01-15 distance post); 22.1151°N, 113.5644°E, ca. 80 m; 11 April 2019; F. Brassard leg.; Winkler; MAC_S11_LLSP_Sp.4; IBBL.

### Key of the twenty-nine *Strumigenys* species recorded from the Guangdong-Hong Kong-Macao Greater Bay Area

The following key relies heavily on couplets elaborated by Bolton (2000), which were subsequently used for a key to the *Strumigenys* (as *Pyramica*) of China (Xu and Zhou 2004). For species present in the Greater Bay Area that are within Bolton’s key, we used his couplets. New couplets were added for species absent from Bolton’s key.

**Table:** 

1	Mandibles relatively short, not kinetic, not forming a snapping mechanism (Fig. 14A). Maximum opening of mandibles 90° or less (17 spp.)	**2**
–	Mandibles relatively elongate, edentate along inner margin and forming a snapping mechanism (i.e., trap-jaw) (Fig. 14B). If not elongate, mandibles forming a snapping mechanism, either curvilinear (Fig. 14C) or with outer margin flared outwards near base and with strongly projecting basal angle (Fig. 14D). Maximum opening of mandibles 170° or more (12 spp.)	**18**
2	Antenna with 4 segments (Fig. 15A); first funicular segment not separated from the 2-segmented apical club (2 spp.)	**3**
–	Antenna with 6 segments (Fig. 15B); first funicular segment separated from the 2-segmented apical club by one or two small segments (15 spp.)	**4**
3	Vertexal corners prominent (Figs 16, 18A). Pilosity on head, mesosoma, petiole, potspetiole and legs consisting of large appressed spatulate hairs (*Native*. China: Hong Kong)	***S. lantaui***
–	Vertexal corners less prominent (Figs 17, 18B). Pilosity on head, mesosoma, petiole and postpetiole consisting of finer erect spatulate hairs. Pilosity on legs consisting of appressed simple hairs (*Native.* China: Yunnan; Northern India, Malaysia, Nepal, Singapore, Thailand, Vietnam. *Introduced*. China: Hong Kong, Macao; Mascarene Islands, India: Kerala)	***S. nepalensis***
4	With head in full-face view, the leading edge of the scape with a row of conspicuous projecting curved hairs, of which one or more, distal to the subbasal bend, distinctly curved toward the base of the scape (Fig. 19A). These hairs may be spatulate, remiform, spoon-shaped or broadly clavate apically; basal stem of each hair (which may be short) erect or sub-erect with respect to the long axis of the scape (4 spp.)	**5**
–	With head in full-face view the leading edge of the scape lacking projecting hairs that curve toward the base of the scape (Fig. 19B). Scape edge may have elongate simple straight projecting hairs present, or entirely apically directed short hairs that may be simple, narrowly to broadly spatulate, or spoon-shaped; in some species the leading edge may be hairless (11 spp.)	**8**
5	Vertexal margin strongly concave (Figs 20, 21A). Vertexal corners forming two conspicuous protrusions. In full-face view, appressed spatulate hairs solely bordering the upper half of clypeus	***S. formosa***
–	Vertexal margin weakly concave (Fig. 21B). Vertexal corners not forming two conspicuous protrusions. In full-face view appressed spatulate hairs or simple hairs found across the whole head, not just on the clypeal margin (3 spp.)	**6**
6	Pilosity on head consisting of spatulate hairs. Eye composed of a single ommatidium (Fig. 22A) (2 spp.)	**7**
–	Pilosity on head consisting of small appressed simple hairs. Eye composed of more than one ommatidium (Figs 22B, 23) (*Native*. Ghana, Sierra Leone, South Africa. *Introduced*. widespread, including China: Guangdong, Hong Kong, Fujian, Macao, Sichuan, Yunnan)	***S. membranifera***
7	Two pairs of thin remiform hairs on the vertex, with one pair on the lateral portions of vertex and the other in posteromedial position (Fig. 24B). Dorsum of promesonotum with erect simple hairs (Fig. 24C) (*Native*: Macao)	***S. subterranea* sp. nov.**
–	Pilosity on head consisting solely of appressed spatulate hairs (Fig. 25A), without erect simple hairs. Dorsum of pronotum with appressed spatulate hairs (Fig. 25B). (*Native*: Guangdong)	***S. lachesis***
8	Petiole node in profile long and relatively flat (Fig. 26A). Petiole node in dorsal view long and narrow (3 spp.)	**9**
–	Petiole node in profile short and with a dorsal protrusion (Fig. 26B). Petiole node in dorsal view short and broad (8 spp.)	**11**
9	Mesopleuron and metapleuron smooth and shiny (Figs 27, 28A). Pilosity on dorsum of mesosoma and posterior margin of head consisting of reduced and appressed simple hairs. Pilosity on first gastral segment short. Propodeal lamella with a thin layer of spongiform tissue (*Native*. China: Hong Kong)	***S. nathistorisoc***
–	Mesopleuron and metapleuron sculptured (Fig. 28B). Pilosity on dorsum of mesosoma and posterior margin of head consisting of long and erect simple hairs. Pilosity of first gastral segment consisting of long and erect simple hairs. Spongiform tissue on propodeal lamina prominent	**10**
10	Dorsum of pronotum with distinct transverse striations and without a median long stria (see Zhou 2011) (*Native*. China: Guangdong)	***S. nankunshana***
–	Dorsum of pronotum without transverse striations and with a median long stria (Fig. 29C) (China: Guangdong, Guangxi, Hong Kong, Macao, Taiwan; Thailand)	***S. elegantula***
11	Dorsal (outer) surfaces of middle and hind tibiae with one or more conspicuous freely laterally projecting long hairs that are at a right-angle or near right-angle to the long axis of the segment (Fig. 30A); these hairs may be straight, curved or flagellate; one or more similar hairs present on basitarsi (2 spp.)	**12**
–	Dorsal (outer) surfaces of middle and hind tibiae and basitarsi with small simple to spatulate decumbent or appressed hairs (Fig. 30B), or with minute appressed pubescence only; lacking freely laterally projecting long hairs that are at a right-angle or near right-angle to the long axis of the segment (6 spp.)	**13**
12	Cuticle on side of head within the scrobe smooth and shining. Dorsal part of mesosoma smooth and shining. Eye with a single ommatidium (Fig. 31) (China: Guangxi, Hong Kong, Taiwan; Japan)	***S. mazu***
–	Cuticle on side of head within the scrobe reticulate-punctate. Dorsum of mesosoma sculptured. Eye with more than one ommatidium (Fig. 32) (Bhutan, China: Fujian, Hunan, Yunnan, Hong Kong)	***S. kichijo***
13	With head in full-face view the entire dorsum clothed with ground pilosity of very conspicuous pale orbicular hairs (Fig. 33A) (2 spp.)	**14**
–	With head in full-face view the dorsum either without hairs or with ground pilosity of short hairs that are simple to narrowly spatulate and usually inconspicuous (Fig. 33B) (4 spp.)	**15**
14	Apical half of mandible with two preapical teeth, the proximal slightly longer than the distal. With alitrunk in profile posterior surface of mesonotum narrowly convex and weakly bulging, overhanging the metanotal groove. Posterodorsal corner of propodeum dentate. Head broader than long (Fig. 34) (*Native*. China: Taiwan; Japan: mainland and Ryukyu Islands ; South Korea. *Introduced*. China: Hong Kong; Japan: Ogasawara Islands; United States of America)	***S. hexamera***
–	Apical half of mandible with a single small inconspicuous preapical tooth, located very close to the spiniform apicodorsal tooth. With alitrunk in profile mesonotum meets propodeum at the metanotal groove, the former not narrowly convex nor bulging posteriorly, not overhanging the metanotal groove. Posterodorsal corner of propodeum rounded. Head slightly longer than broad (Fig. 35) (China: Hong Kong, Guangdong, Hubei, Hunan)	***S. tisiphone***
15	With head in full-face view, the outer margins of the fully closed mandibles intersect the anterior clypeal margin mesad of the anterolateral clypeal angles, so that there is a section of the anterior clypeal margin that projects laterally beyond the outer line or the mandible (Fig. 36A). Small to minute species (TL: > 2 to 3 mm) (2 spp.)	**16**
–	With head in full-face view, the outer margins of the fully closed mandibles intersect the anterior clypeal margin at the anterolateral clypeal angles, so that there is no section of the anterior clypeal margin that projects laterally beyond the outer line of the mandible (Fig. 36B). Minute species (TL < 2 mm) (2 spp.)	**17**
16	Anterior clypeal margin shallowly transversely concave across its entire width (Fig. 37A). Mandible with 14 teeth distal of a long low basal lamella (China: Hong Kong, Macao; Japan, South Korea, Vietnam)	***S. canina***
–	Anterior clypeal margin with a deep semicircular median impression, the anterolateral angles broadly convex on each side of the impression (Fig. 38A). Mandible with 12 teeth distal of a triangular rounded basal lamella (China: Fujian, Guangxi, Hong Kong, Hunan, Macao, Taiwan, Yunnan; Japan, Thailand)	***S. sauteri***
17	With head in full-face view, the fully closed mandibles triangular, with teeth present along entire length of exposed inner margin (Fig. 39A); proximal half or inner margin dentate, without a long diastema between basal tooth and basal lamella; without a large space basally through which the apices of the labral lobes are visible (Brunei Darussalam, China, India, Indonesia, Malaysia, New Guinea, Philippines, Thailand, Vietnam)	***S. mitis***
–	With head in full-face view, the fully closed mandibles narrow or elongate-triangular, with teeth present only on distal half of exposed length of inner margin (Fig. 40A); proximal half of inner margin edentate and forming a long diastema between basal tooth and basal lamella; a large space present basally between the opposed mandibles through which the apices or the labral lobes are visible (China: Guangxi, Hunan, Hong Kong; Japan, South Korea, Taiwan)	***S. mutica***
18	Antenna with 4 segments (Figs 41, 42A); first funicular segment not separated from the 2-segmented apical club (Pantropical distribution)	***S. emmae***
–	Antenna with 6 segments (Fig. 42B); first funicular segment separated from the 2-segmented apical club by two small segments (11 spp.)	**19**
19	Fully closed mandibles in full-face view very broad proximally and strikingly tapered distally, obviously not linear or curvilinear (Figs 43, 44C). Outer margin of mandible flared outwards near base and with a strongly projecting prebasal angle (China: Hong Kong ; Indonesia: Java ; Thailand, Vietnam)	***S. sydorata***
–	Fully closed mandible in full-face view not very broad proximally nor strikingly tapered distally, linear (Fig. 44A) or curvilinear (Fig. 44B). Outer margin of mandible not flared outwards near base, without a strongly projecting prebasal angle (10 spp.)	**20**
20	Preapical dentition of each mandible with 2 preapical teeth (Fig. 45A) (*Native*: Afrotropical region ; *Introduced*: widespread)	***S. rogeri***
–	Preapical dentition of each mandible either absent or of a single article; when present with either a single tooth or a single denticle (9 spp.)	**21**
21	With head in full-face view mandible without preapical dentition (Figs 46A, 47A), no trace of a projecting preapical tooth or denticle (China: Hong Kong)	***S. heteropha***
–	With head in full-face view mandible with preapical dentition, a projecting preapical tooth (Fig. 47B) or denticle present (8 spp.)	**22**
22	With mesosoma in profile the propodeal declivity equipped with a broad and conspicuous spongiform lamella (Fig. 48A); the propodeal tooth may be replaced by the lamella or completely buried in the lamella, or lamella may subtend the ventral margin of the tooth for most or all of its length (3 spp.)	**23**
–	With mesosoma in profile view the propodeal declivity equipped with a simple carina or at most a narrow cuticular flange (Fig. 48B); carina or narrow flange does not subtend the ventral margin of the tooth for most or all of its length (5 spp.)	**24**
23	Pronotal humeral hair stiff, straight, relatively short (Figs 49, 50A) (Guangdong, Taiwan)	***S. hispida***
–	Pronotal humeral hair flagellate, long and slender (Fig. 50B) (2 spp.)	**25**
24	Dorsal surface of petiole node and disc of postpetiole both smooth and shining, the two surfaces not contrasting. With petiole in dorsal view the node without a truncated anterior face. Smaller ant (TL = 2), with shorter head (HL = 0.52–0.54) and antennae (SL = 0.28–0.30) (Figs 51, 53A) (China: Hong Kong, Macao, Taiwan; Japan)	***S. minutula***
–	Dorsal surface of petiole node sharply punctate or reticulate-punctate, disc of postpetiole smooth or with very scattered faint sculptural vestiges, the two surfaces contrasting. With petiole in dorsal view the node with a short truncated anterior face; lateral margins not converging to a triangular anteromedian point. Larger ant (TL = 2.2–2.6), with longer head (HL = 0.58–0.71) and antennae (SL = 0.34–0.42) (Figs 52, 53B) (Bhutan, China: Hong Kong, Taiwan; India, Indonesia, Malaysia, Thailand)	***S. nanzanensis***
25	Preapical tooth of mandible spiniform and shallowly curved (Figs 54, 55A), its length ca. one-third greater than the maximum width of the mandible (China: Hong Kong; Vietnam)	***S. rallarhina***
–	Preapical tooth of mandible varying from a denticle to a triangular tooth but not spiniform, the tooth shorter than the maximum width of the mandible, usually distinctly shorter (Fig. 55B) (4 spp.)	**26**
26	Preapical tooth very small, in full-face view its length one-quarter or less of the width of the mandible at the point where the tooth arises (Figs 56, 57A) (China: Hong Kong, Macao; Myanmar, Indomalaya)	***S. feae***
–	Preapical tooth larger, in full-face view its length half or more of the width of the mandible at the point where the tooth arises (Fig. 57B) (3 spp.)	**27**
27	In full-face view, external margin of mandibles straight (Figs 58, 59A) (China: Guangdong; Japan)	***S. stenorhina***
–	In full-face view, external margin of mandibles curvilinear (Fig. 59B) (2 spp.)	**28**
28	In full-face view, long appressed simple hairs abundant on head, antennae and mandibles (Fig. 60A). Metapleuron partly smooth, with the majority of its surface punctate (*Native*. China: Hong Kong)	***S. hirsuta***
–	In full-face view, pilosity on head and antennae consisting mostly of relatively slender appressed spatulate hairs (Fig. 61A). Pilosity on mandibles consisting of short appressed simple hairs. Metapleuron completely smooth and shining (Bhutan, China: Hong Kong, Macao; India, Japan, Nepal, Thailand)	***S. exilirhina***

**Figure 14.**
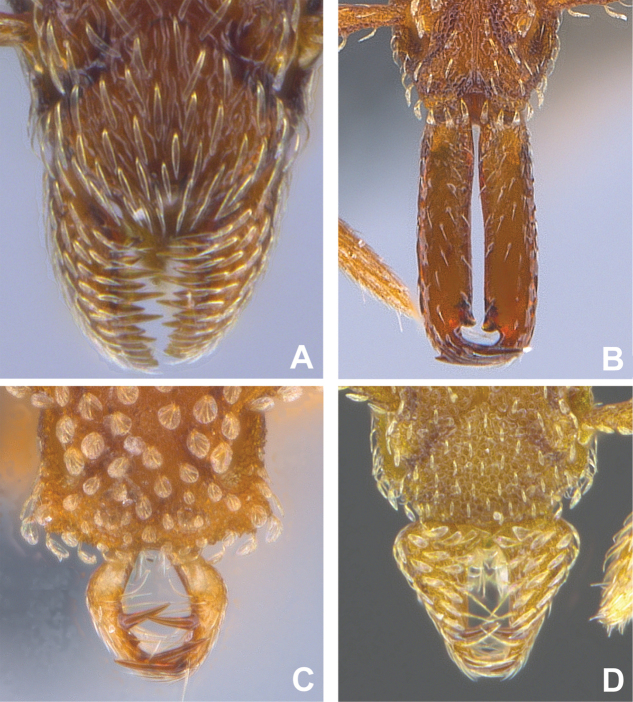
Examples of short, not kinetic, mandibles in *S. elegantula* (**A** MAC_S04_LLSP_sp.9, photograph by IBBL), relatively long and kinetic in *S. feae* (**B** MAC_S15_LLSP_sp.8, photograph by IBBL), curvilinear and kinetic in *S. emmae* (**C** MAC_S20_LLSP_sp.7, photograph by IBBL), and with outer margin flared outwards near base and with strongly projecting basal angle in *S. sydorata* (**D** RHL003404, photograph by IBBL).

**Figure 15.**
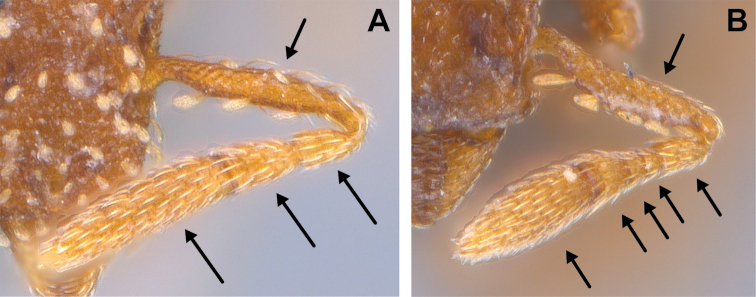
Examples of antenna with 4 segments in *S. nepalensis* (**A** MAC_S19_LLSP_sp.3, photograph by IBBL) and antenna with 6 segments in *S. membranifera* (**B** MAC_S21_LLSP_sp.2, photograph by IBBL).

**Figure 16.**
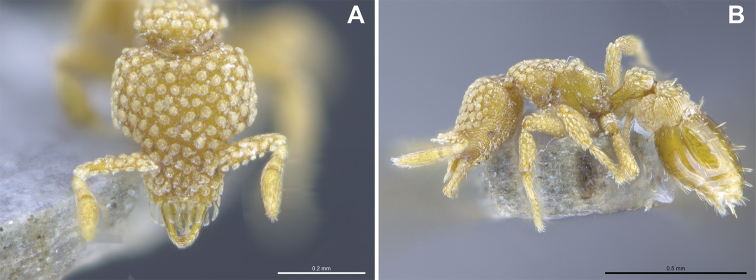
*Strumigenys lantaui* (ANTWEB1009620, photographed by IBBL) in full-face **A** and profile view **B**.

**Figure 17.**
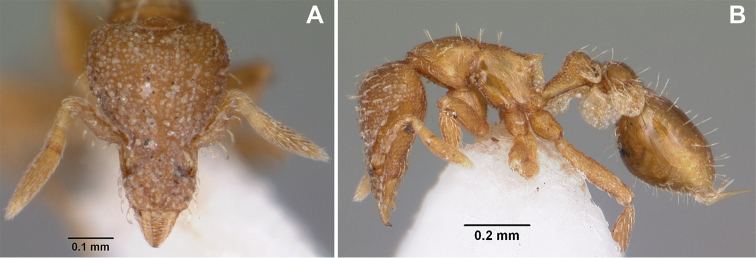
*Strumigenys nepalensis* (ANTWEB0102623, photographed by April Nobile) in full-face **A** and profile view **B**.

**Figure 18.**
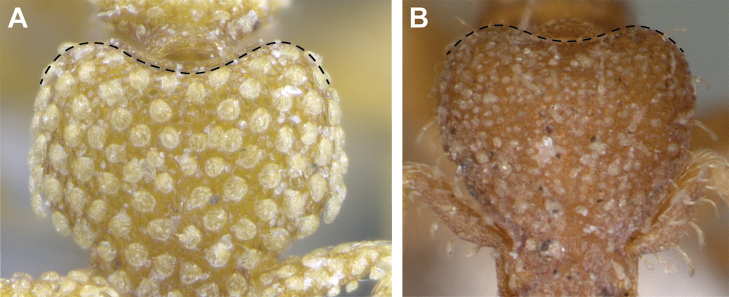
Examples of prominent vertexal corners in *S. lantaui* (**A** ANTWEB1009620, photographed by IBBL) and less prominent vertexal corners in *S. nepalensis* (**B** ANTWEB0102623, photographed by April Nobile).

**Figure 19.**
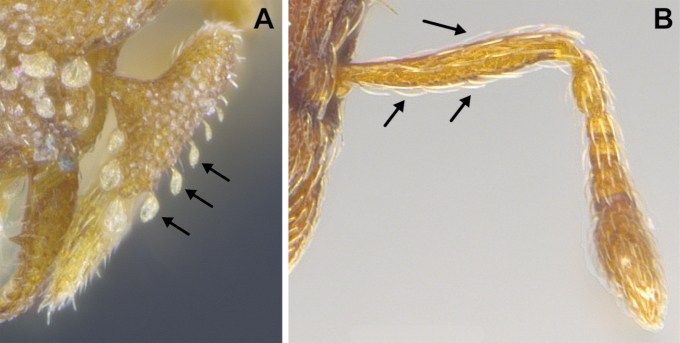
Examples of leading edge of scape with conspicuous hairs curving towards the base of the scape in *S. formosa* (**A** RHL003476, photographed by IBBL) and of leading edge of scape lacking projecting hairs that curve toward the base of the scape in *S. elegantula* (**B** MAC_S04_LLSP_sp.9, photographed by IBBL).

**Figure 20.**
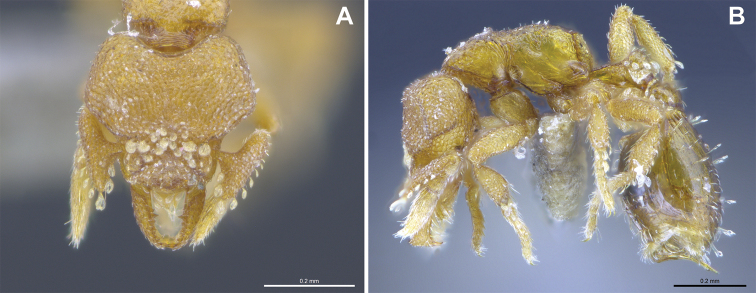
*Strumigenys formosa* (RHL003476, photographed by IBBL) in full-face **A** and profile view **B**.

**Figure 21.**
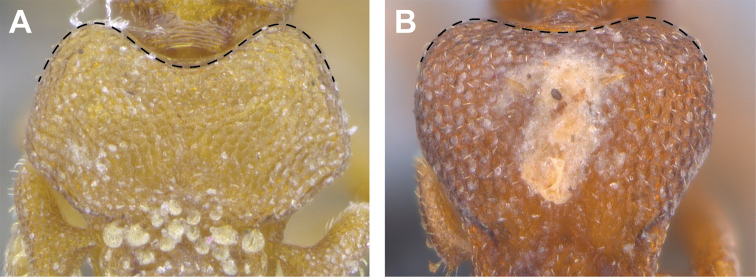
Examples of vertexal margin strongly concave in *S. formosa* (**A** RHL003476, photographed by IBBL) and of vertexal margin weakly concave in *S. membranifera* (**B** MAC_S21_LLSP_Sp.2, photographed by IBBL).

**Figure 22.**
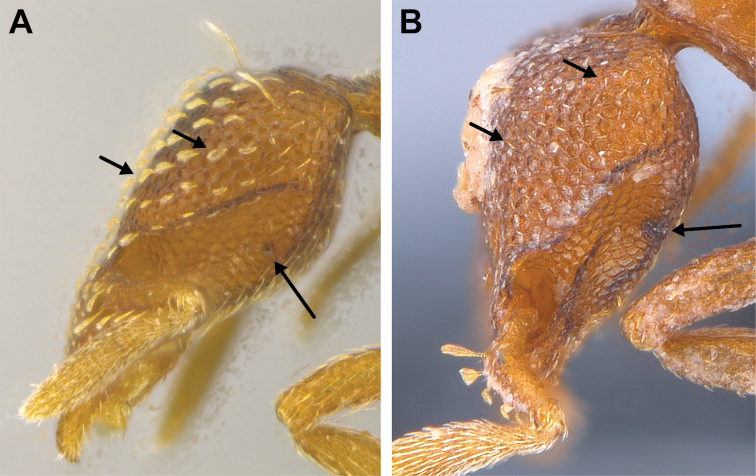
Examples of spatulate hairs on head and eye with a single ommatidium in *S. subterranea* sp. nov. (**A** ANTWEB1010847, photographed by François Brassard) and of small appressed simple hairs on head with eye composed of more than one ommatidium in *S. membranifera* (**B** MAC_S21_LLSP_sp.2, photographed by IBBL).

**Figure 23.**
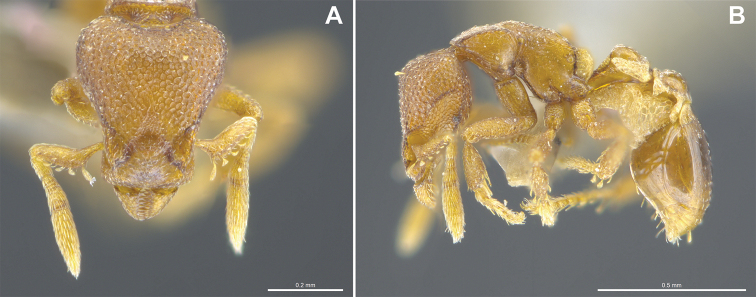
*Strumigenys membranifera* (BMW02021, photographed by IBBL) in full-face **A** and profile view **B**.

**Figure 24.**
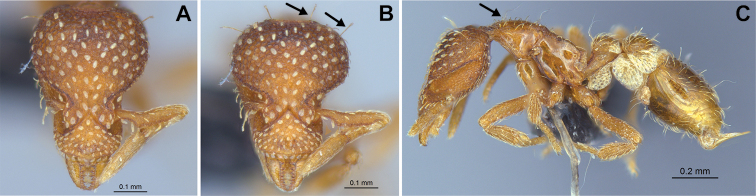
*Strumigenys subterranea* (ANTWEB1010847, photographed by François Brassard) in full-face **A, B** and profile view **C**.

**Figure 25.**
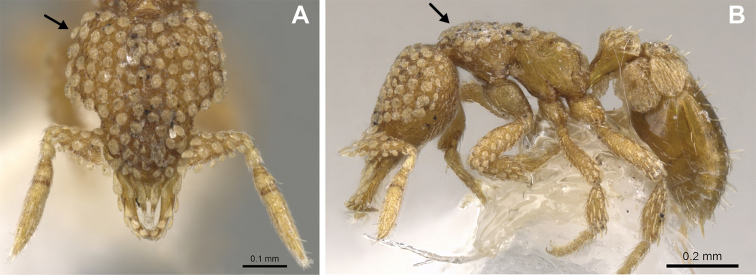
*Strumigenys lachesis* (ANTWEB0900156, photographed by Will Ericson) in full-face **A** and profile view **B**.

**Figure 26.**
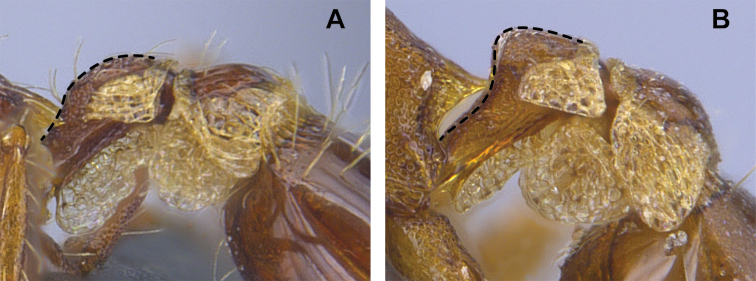
Examples of long and relatively flat petiole in *Strumigenys elegantula* (**A** ; MAC_S04_LLSP_sp.9), and short with a dorsal protrusion in *Strumigenys sauteri* (**B** ; MAC_S04_LLSP_sp.2).

**Figure 27.**
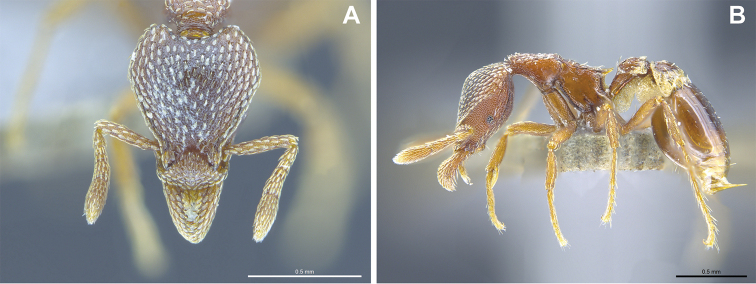
*Strumigenys nathistorisoc* (ANTWEB1016948, photographed by IBBL) in full-face **A** and profile view **B**.

**Figure 28.**
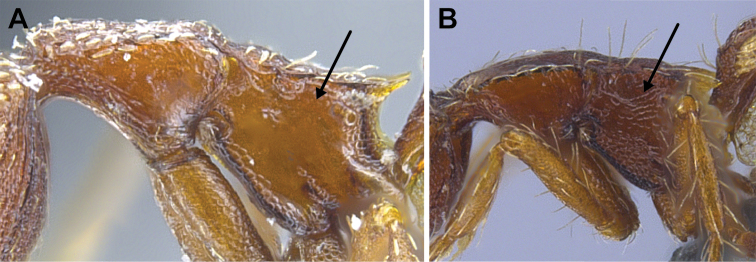
Examples of smooth and shiny mesopleuron and metapleuron in *S. nathistorisoc* (**A** ANTWEB1016948, photographed by IBBL), and of sculptured mesopleuron and metapleuron in *S. elegantula* (**B** MAC_S04_LLSP_sp.9, photographed by Siu Yiu).

**Figure 29.**
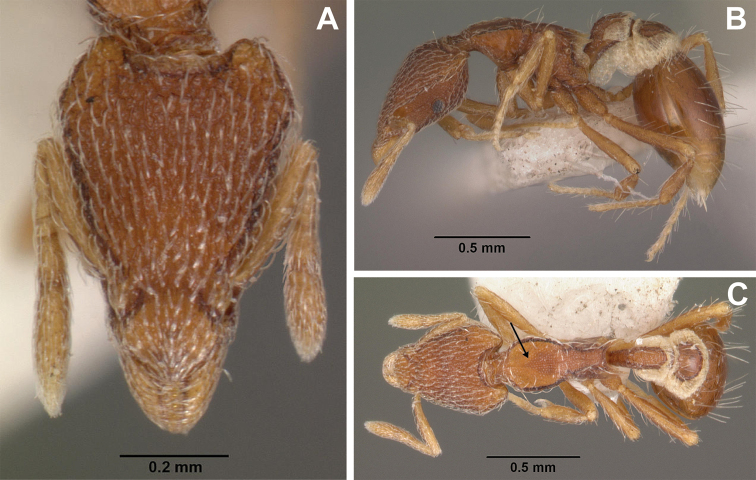
*Strumigenys elegantula* (ANTWEB0102542, photographed by April Nobile) in full-face **A**, profile **B**, and dorsal view **C**.

**Figure 30.**
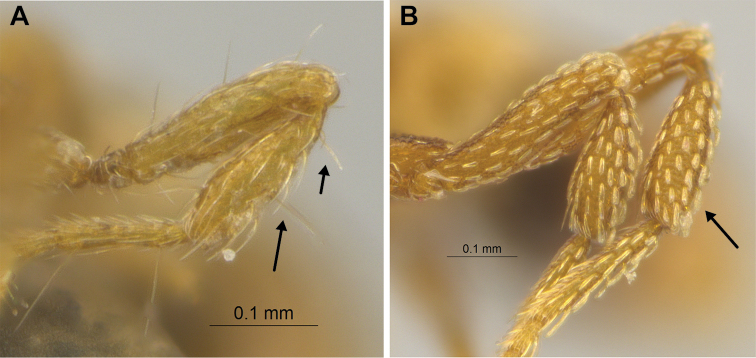
Examples of conspicuous laterally projecting hairs on middle and hind tibiae *S. mazu* (**A** ANTWEB1017070, photographed by IBBL) and of small appressed hairs in *S. tisiphone* (**B** RHL02818, photographed by IBBL).

**Figure 31.**
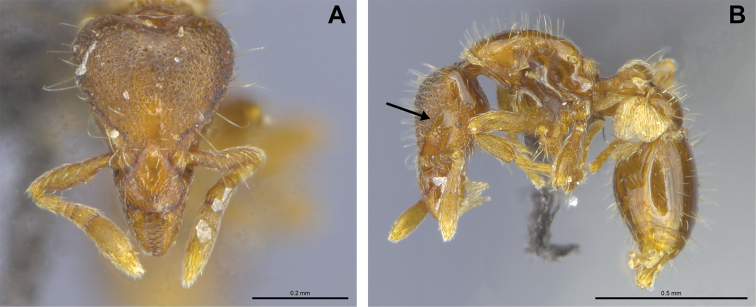
*Strumigenys mazu* (TT00985, photographed by IBBL) in full-face **A** and profile view **B**.

**Figure 32.**
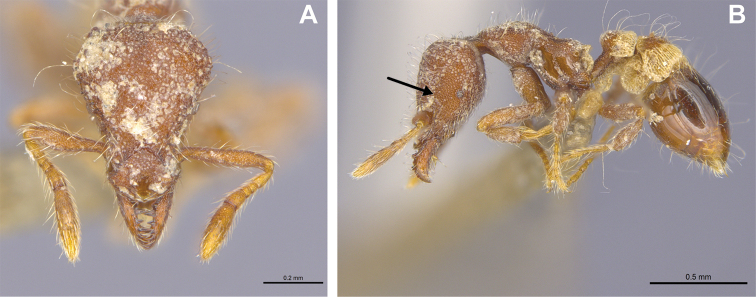
*Strumigenys kichijo* (RHL003471, photographed by IBBL) in full-face **A** and profile view **B**.

**Figure 33.**
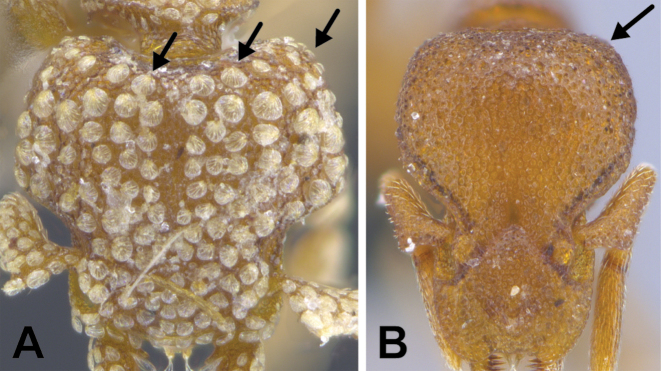
Examples of head with dorsum clothed with ground pilosity of conspicuous pale orbicular hairs in *S. hexamera* (**A** RHL003477, photographed by IBBL) and of dorsum of head without hairs in *S. sauteri* (**B** MAC_S04_LLSP_sp.2, photographed by IBBL).

**Figure 34.**
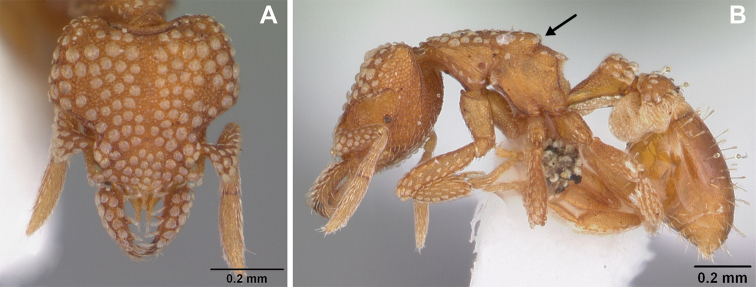
*Strumigenys hexamera* (ANTWEB0103819, photographed by April Nobile) in full-face **A** and profile view **B**.

**Figure 35.**
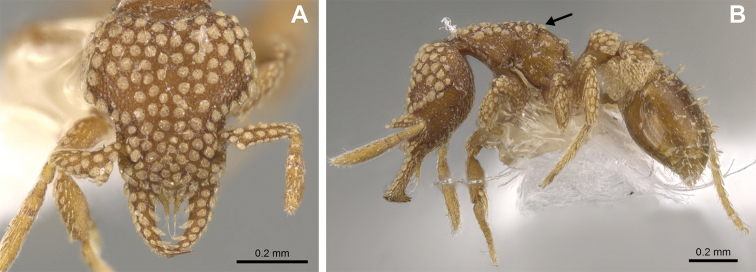
*Strumigenys tisiphone* (ANTWEB0900154, photographed by Will Ericson) in full-face **A** and profile view **B**.

**Figure 36.**
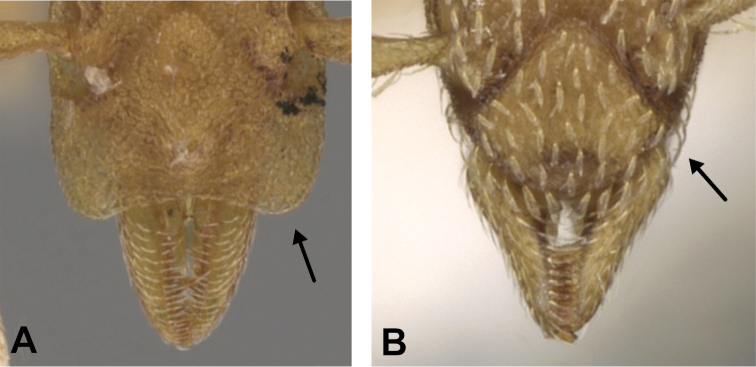
Examples of anterior clypeal margin projecting laterally beyond the outer line of the mandible in *S. canina* (**A** ANTWEB0900124, photographed by Will Ericson) and of anterior clypeal margin not projecting beyond the outer line of the mandible in *S. mutica* (**B** ANTWEB0280715, photographed by Shannon Hartman).

**Figure 37.**
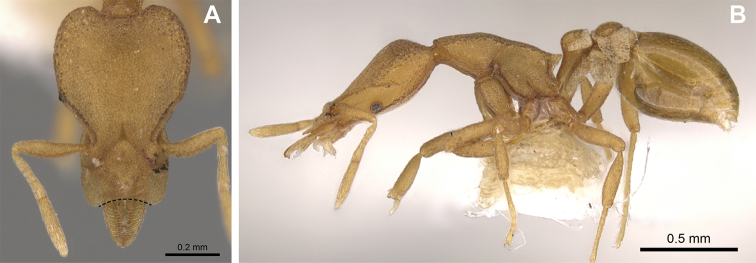
*Strumigenys canina* (ANTWEB0900124, photographed by Will Ericson) in full-face **A** and profile view **B**.

**Figure 38.**
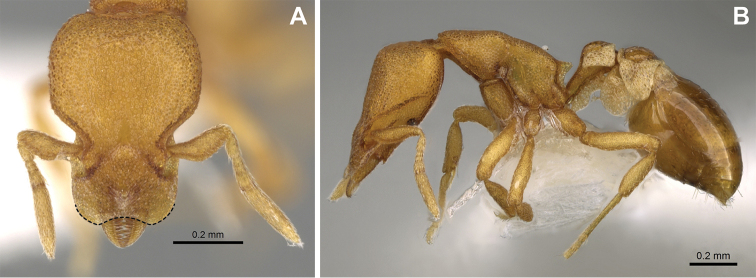
*Strumigenys sauteri* (ANTWEB0280702, photographed by Shannon Hartman) in full-face **A** and profile view **B**.

**Figure 39.**
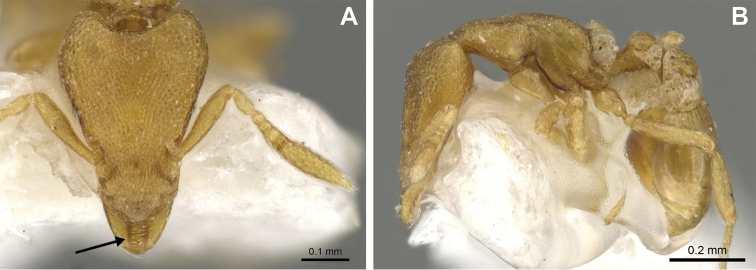
*Strumigenys mitis* (ANTWEB0900120, photographed by Will Ericson) in full-face **A** and profile view **B**.

**Figure 40.**
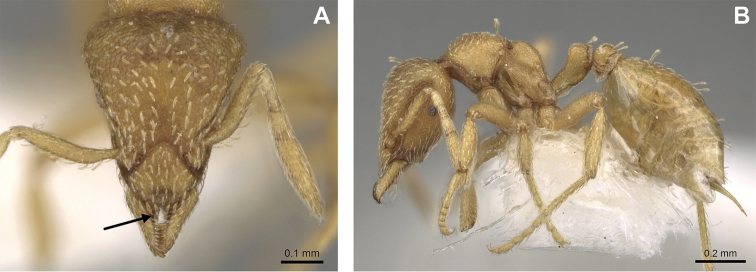
*Strumigenys mutica* (ANTWEB0280715, photographed by Shannon Hartman) in full-face **A** and profile view **B**.

**Figure 41.**
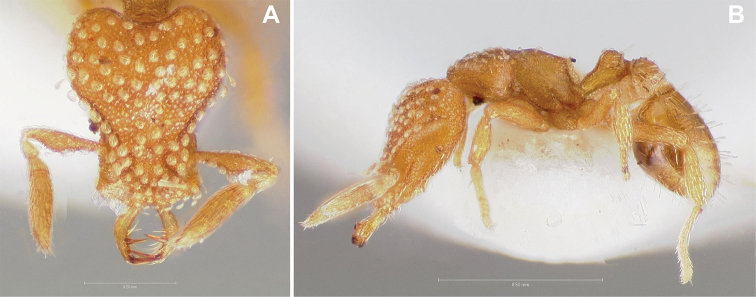
*Strumigenys emmae* (CASENT000589, photographed by April Nobile) in full-face **A** and profile view **B**.

**Figure 42.**
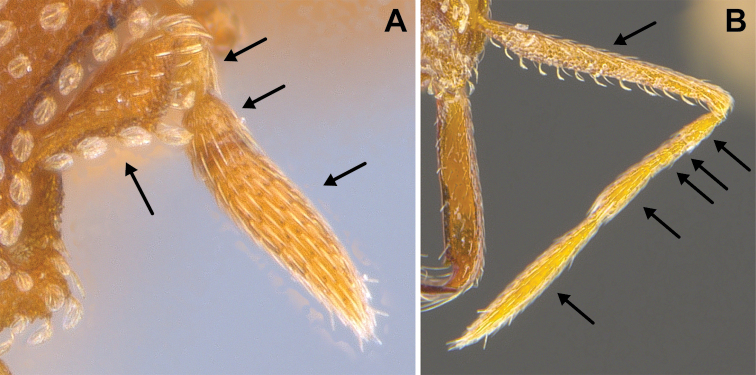
Examples of antenna with 4 segments in *S. emmae* (**A** MAC_S20_LLSP_sp.7, photographed by IBBL) and of antenna with 6 segments in *S. feae* (**B** ANTWEB1017082, photographed by IBBL).

**Figure 43.**
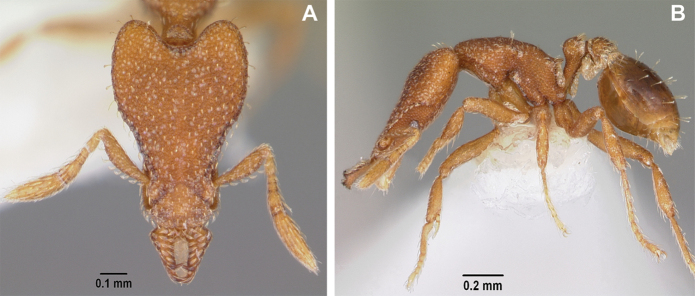
*Strumigenys sydorata* (ANTWEB0102619, photographed by April Nobile) in full-face **A** and profile view **B**.

**Figure 44.**
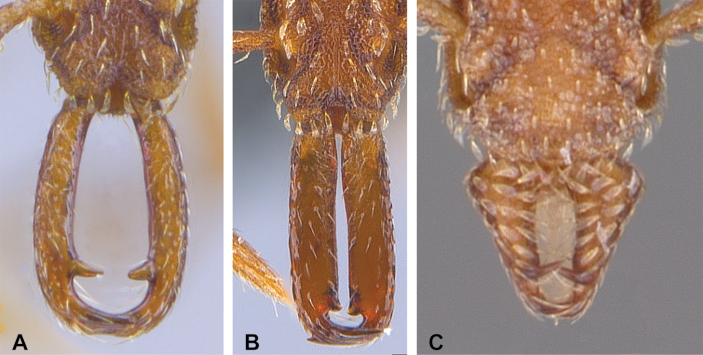
Examples of mandibles of different shape: linear mandibles in *S. minutula* (**A** MAC_S01_LLSA_Sp.3, photographed by François Brassard), curvilinear mandibles in *S. feae* (**B** MAC_S15_LLSP_Sp.8, photographed by François Brassard), and broad proximally and mandibles strikingly tapered distally in *S. sydorata* (**C** ANTWEB0102619, photographed by April Nobile).

**Figure 45.**
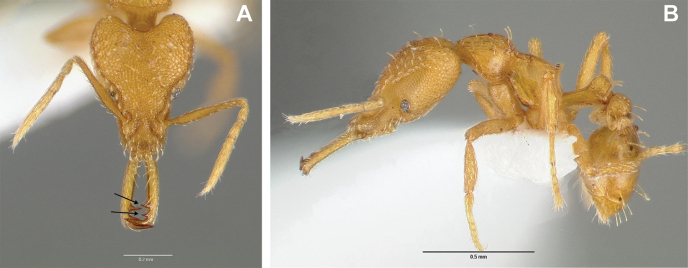
*Strumigenys rogeri* (CASENT0005897, photographed by April Nobile) in full-face **A** and profile view **B**.

**Figure 46.**
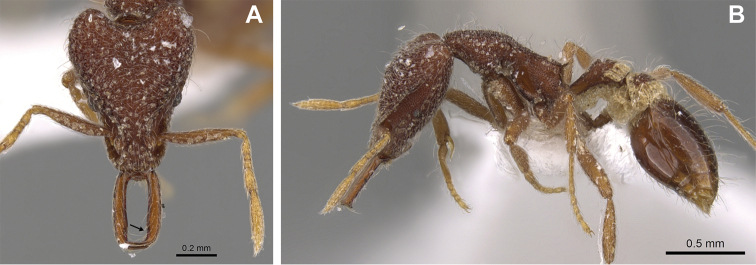
*Strumigenys heteropha* (CASENT0005897, photographed by Will Ericson) in full-face **A** and profile view **B**.

**Figure 47.**
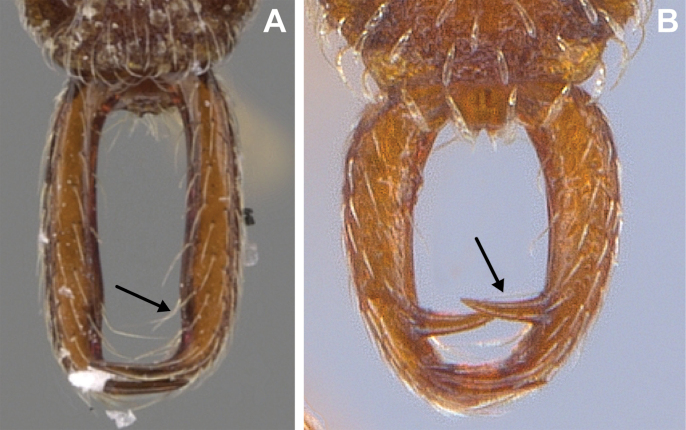
Examples of mandible without preapical dentition in *S. heteropha* (**A** CASENT CASENT0005897, photographed by Will Ericson) and of mandible with preapical dentition in *S. minutula* (**B** MAC_S14_LLSP_sp.4, photographed by François Brassard).

**Figure 48.**
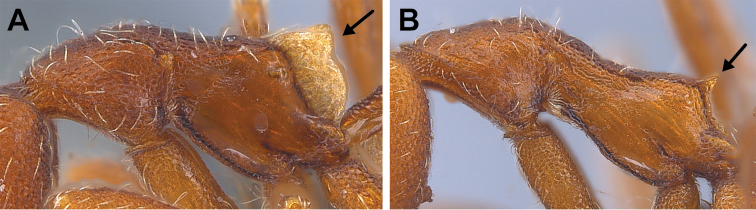
Propodeal declivity equipped with a broad and conspicuous lamella (**A** MAC_S14_LLSP_Sp.4, photographed by François Brassard) and propodeal declivity equipped with a simple carina in *S. feae* (**B** MAC_S15_LLSP_Sp.8, photographed by François Brassard).

**Figure 49.**
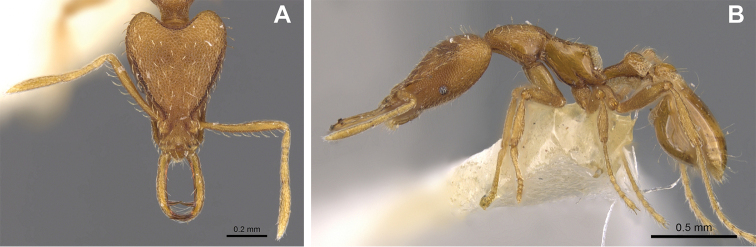
*Strumigenys hispida* (CASENT0900821, photographed by Will Ericson) in full-face **A** and profile view **B**.

**Figure 50.**
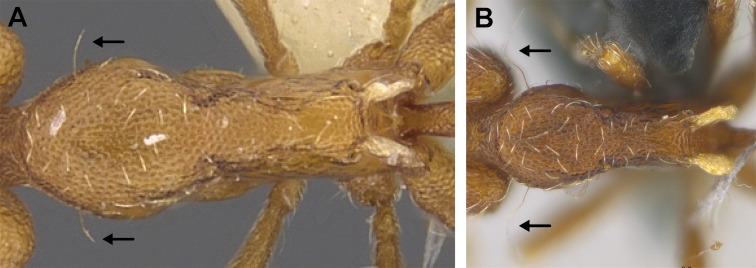
Examples of relatively short and straight, stiff pronotal humeral hairs in *S. hispida* (**A** CASENT0900821, photographed by Will Ericson) and of long and slender flagellate pronotal hairs in *S. minutula* (**B** MAC_LLSA_S06_sp.6, photographed by François Brassard).

**Figure 51.**
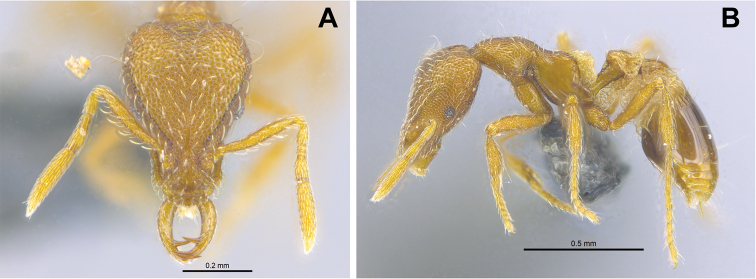
*Strumigenys minutula* (MAC_S11_GN3_H4_n1, photographed by François Brassard) in full-face **A** and profile view **B**.

**Figure 52.**
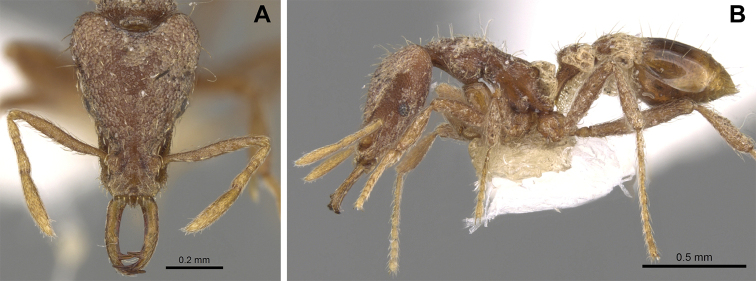
*Strumigenys nanzanensis* (CASENT02807, photographed by Shannon Hartman) in full-face **A** and profile view **B**.

**Figure 53.**
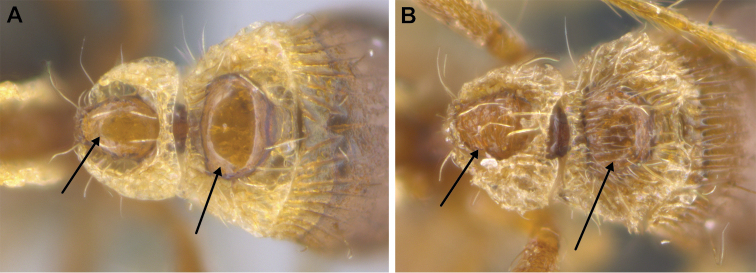
Examples of smooth dorsal surface of petiole node and disc of postpetiole in *S. minutula* (**A** MAC_LLSA_S06_sp.6, photographed by François Brassard) and of reticulate-punctate dorsal surface of petiole node and postpetiole smooth with very scattered faint sculptural vestiges in *S. nanzanensis* (**B** BMW00846, photographed by François Brassard).

**Figure 54.**
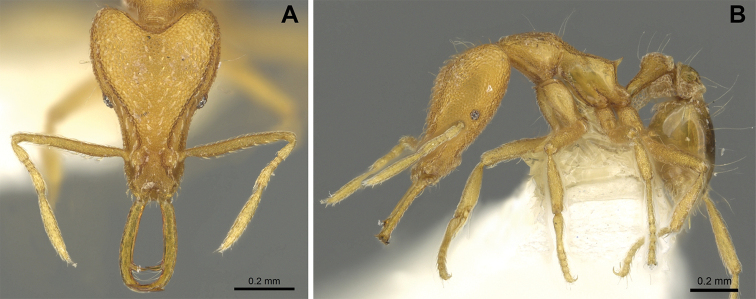
*Strumigenys rallarhina* (CASENT0900839, photographed by Ryan Perry) in full-face **A** and profile view **B**.

**Figure 55.**
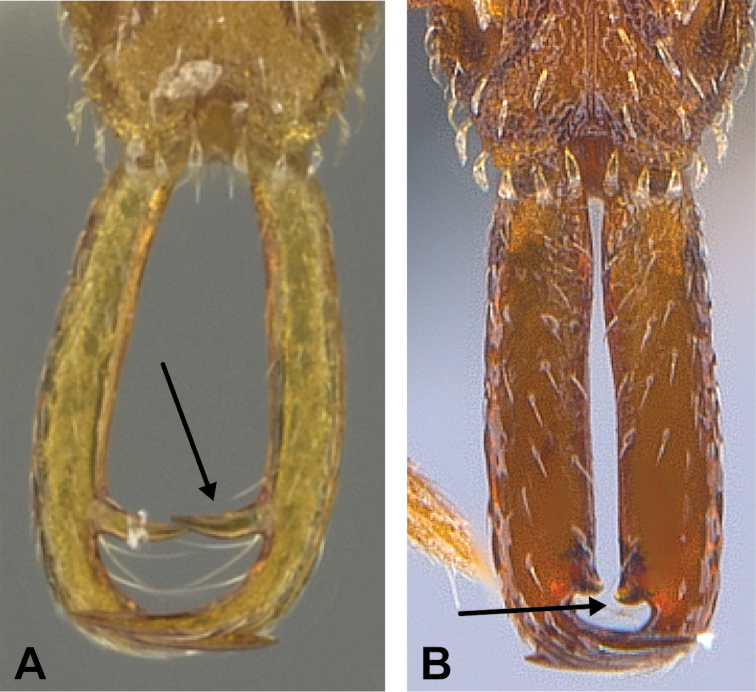
Example of spiniform and shallowly curved preapical tooth in *S. rallarhina* (**A** CASENT0900839, photographed by Ryan Perry) and of short triangular tooth in *S. feae* (**B** MAC_S15_LLSP_sp.8, photographed by IBBL).

**Figure 56.**
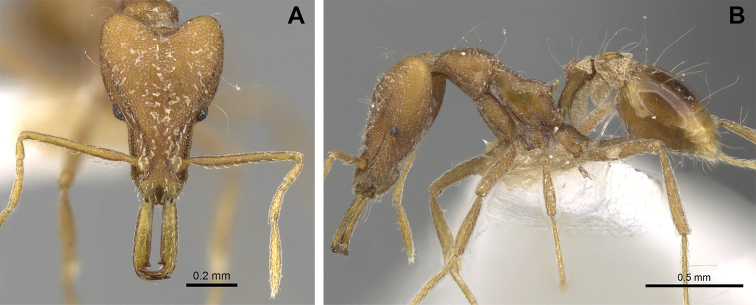
*Strumigenys feae* (CASENT0280748, photographed by Shannon Hartman) in full-face **A** and profile view **B**.

**Figure 57.**
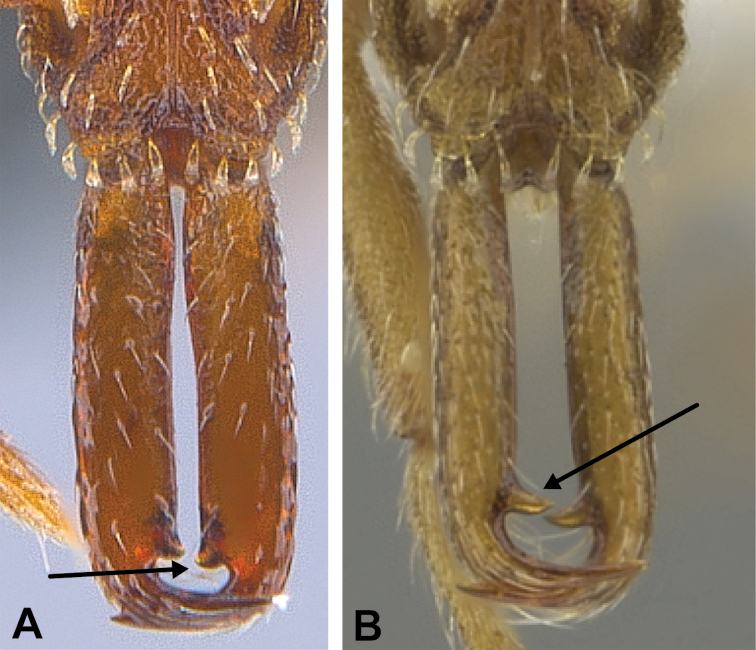
Examples of small preapical tooth in *S. feae* (**A** MAC_S15_LLSP_sp.8, photographed by IBBL) and of larger preapical tooth in *S. stenorhina* (**B** CASENT0900840, photographed by Ryan Perry).

**Figure 58.**
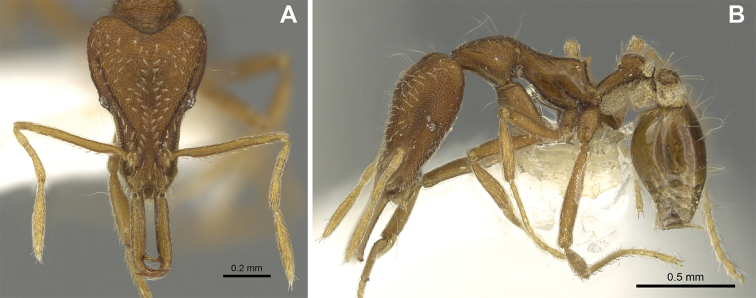
*Strumigenys stenorhina* (CASENT0900840, photographed by Ryan Perry) in full-face **A** and profile view **B**.

**Figure 59.**
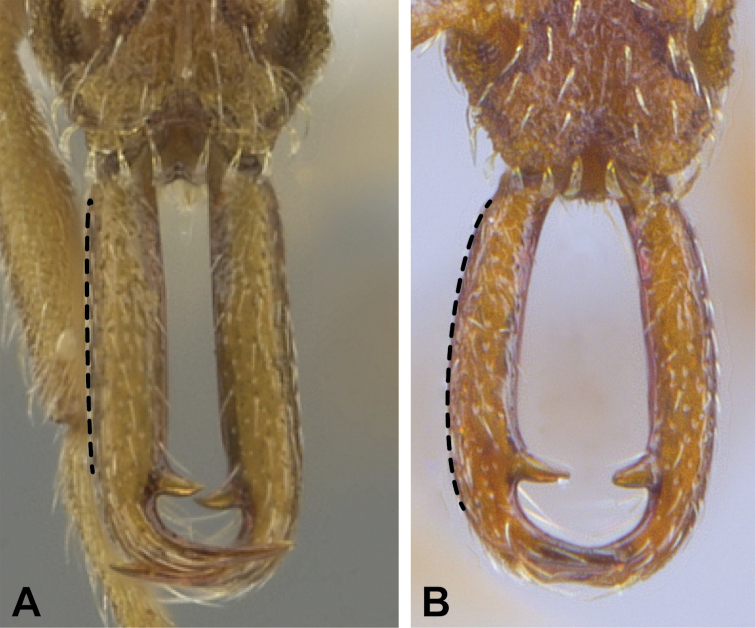
Examples of mandible with straight external margin in *S. stenorhina* (**A** CASENT0900840, photographed by Ryan Perry) and of mandible with curvilinear external margin in *S. exilirhina* (**B** MAC_S01_LLSA_sp.3, photographed by IBBL).

**Figure 60.**
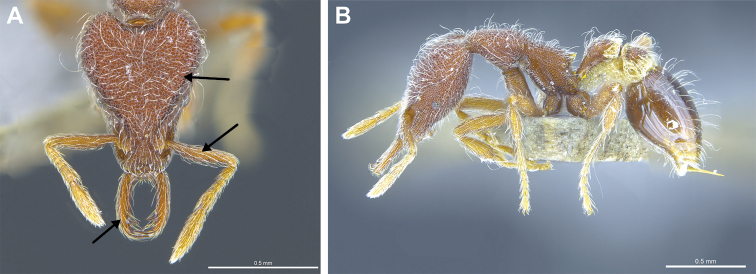
*Strumigenys hirsuta* (ANTWEB1009855, photographed by IBBL) in full-face **A** and profile view **B**.

**Figure 61.**
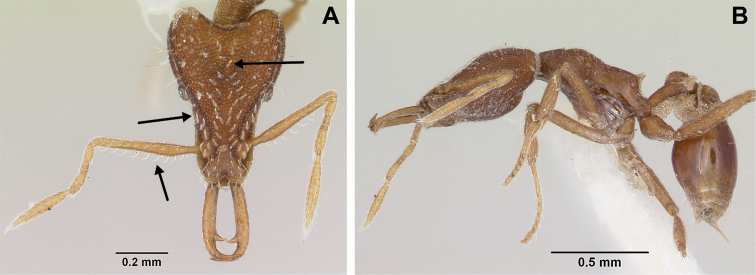
*Strumigenys exilirhina* (CASENT0102650, photographed by April Nobile) in full-face **A** and profile view **B**.

## Discussion

Traditionally, *Strumigenys* species have been collected through the extraction of arthropods present in the leaf litter, which is here confirmed with the capture of eight out of nine species through this method. However, the addition of subterranean traps allowed the collection of an undescribed species: *Strumigenys subterranea* sp. nov. If the majority of *Strumigenys* species are not considered subterranean, but rather leaf-litter foragers or even arboreal (Longino and Nadkarni 1990; Lattke et al. 2018), a few exceptions occur. For instance, the species *Strumigenys hexamera* Brown, 1958 (Masuko 1984) and *Strumigenys mitis* Brown, 2000 (Mezger and Pfeiffer 2010) are considered subterranean, while other records suggest a potential subterranean lifestyle in other species such as in Strumigenys sp. nr. sutrix Bolton, 2000, for which 2 individuals were collected with subterranean traps at a depth of 5 cm (Andersen and Brault 2010).

Subterranean ants have adaptations, such as specific morphological characteristics, to live within the particular environmental conditions that define the underground habitat (Wong and Guénard 2017). Among those, the absence of eyes or the presence of reduced eyes, while not strictly limited to subterranean ants (e.g., some epigaeic army ants), represent an adaptation to this obscure environment. Indeed, visual orientation underground should be limited if non-existent, thus rendering obsolete the need for large eyes providing a more acute vision. The presence of short mandibles, presumably easier to maneuver underground than the long and snapping mandibles of certain trap-jaw *Strumigenys*, potentially represents another adaptation. For instance, *S. hexamera* is a sit and wait predator that uses its short mandibles to hunt preys within tunnels (Masuko 1984), while the short triangular mandibles of the subterranean *S. mitis* have been proposed as a subterranean adaptation in contrast to the long and wide-opening mandibles of the epigaeic *S. rotogenys* (Mezger & Pfeiffer, 2010). Since *Strumigenys subterranea* sp. nov. possesses both characteristics (i.e., reduced eyes and short triangular mandibles) and was detected at 12.5 cm below ground, this would suggest a subterranean lifestyle. Nevertheless, mentions of subterranean *Strumigenys* are still rare, which may be due to very limited sampling efforts within this stratum (Wong and Guénard 2017).

To collect subterranean *Strumigenys*, other techniques than subterranean traps exist. For instance, *Strumigenys louisianae* Roger, 1863, Strumigenys nr. epinotalis Weber, 1934 and *Strumigenys denticulata* Mayr, 1887 were retrieved using soil monoliths at a depth of 0–10cm (Martins et al. 2020), while the holotype worker of *Strumigenys fuarda* Bolton, 2000 was collected within a soil core (Bolton 2000). However, soil sampling, if done without removing the upper soil layer, does not preclude the collection of leaf litter ants foraging on the upper surface of a core or monolith. This is potentially the case for *S. louisianae* or S. nr. epinotalis (Martins et al. 2020), and as such further observations are required to establish if they are hypo- or epigaeic.

Another sampling method that can potentially collect subterranean *Strumigenys* is nest excavation, which has been used to collect nests of the subterranean *S. hexamera* (Masuko, 2013). Moreover, excavations under the litter-fermentation-humidification horizon up to a depth of 25 cm found nests of *Strumigenys kumadori* Yoshimura & Onoyama, 2007 (Masuko 2010). However, nest excavations do not ensure that the species collected are subterranean, because even though these species may nest underground, their foraging activity could be mainly occurring above ground. As such, labeling an ant as subterranean solely because it was collected during an excavation is not fully satisfactory. As an example, *S. kumadori*, which has relatively large eyes and long snapping mandibles, does not match the morphology of an hypogaeic species. Thus, to collect subterranean *Strumigenys* species, subterranean traps or the careful excavation of soil monoliths (of which the top soil layers would be excluded) seem to be preferential solutions.

## Conclusions

Although cities and the nature parks within them (i.e., patches of secondary forests) are rarely viewed as a refuge for biodiversity, recent work using diverse sampling approaches have shown that urban habitats can host high numbers of both native and exotic ant species (Guénard et al. 2015; Leong et al. 2017). This study in Macao, as well as recent work in Hong Kong (Tang et al. 2019) – two heavily urbanized regions – shows that both regions support a high diversity of *Strumigenys* species, with nine and 24 species recorded respectively. Recent work in other parts of the world, such as in the USA, also showed that the discovery of new *Strumigenys* species within urban habitats is possible (Longino and Booher 2019). Alarmingly, further urbanization threatens several of these habitats, including the type locality of the species described here. Indeed, a construction project is planned in proximity of where the only specimen of *Strumigenys subterranea* sp. nov. was found, which could potentially impact key habitats for this species. In summary, the current study supports both the ideas that urban areas can hold a surprisingly high biodiversity for particular taxa as well as to contain species novel to science. As such, it is worth protecting forest patches within cities, and using extensive sampling methods to discover and describe what lurks amongst our dwellings.

## Supplementary Material

XML Treatment for Strumigenys subterranea

XML Treatment for Strumigenys elegantula

XML Treatment for Strumigenys emmae

XML Treatment for Strumigenys exilirhina

XML Treatment for Strumigenys feae

XML Treatment for Strumigenys membranifera

XML Treatment for Strumigenys minutula

XML Treatment for Strumigenys nepalensis

XML Treatment for Strumigenys sauteri

## Supplementary materials

Supplementary material 1 Figure S1. Map of Coloane Island showcasing the 21 sites sampled Data type occurrence Explanation note White dots mark sites where the full protocol was done (i.e., leaf litter extraction, ground baiting, ground nests, subterranean traps and arboreal traps), whereas grey dots mark preliminary sites where only ground baiting and leaf litter extraction were done. Hand collection was also opportunistically used at each site. https://binary.pensoft.net/file/455300This dataset is made available under the Open Database License (http://opendatacommons.org/licenses/odbl/1.0/). The Open Database License (ODbL) is a license agreement intended to allow users to freely share, modify, and use this Dataset while maintaining this same freedom for others, provided that the original source and author(s) are credited.François Brassard, Chi-Man Leong, Hoi-Hou Chan, Benoit Guénard

Supplementary material 2 Figure S2. Schematic representation of the subterranean sampling protocol used in the study Data type measurement Explanation note For a site, 4 different quadrats each had four traps placed 1 m apart at each of their corners (n = 16). Within a quadrat, traps were distributed at four different depths: 12.5, 25, 37.5, and 50 cm (A). Traps consisted of a 15 mL falcon tube containing 70 % ethanol at its bottom (B). To attract ants, a bait consisting of tuna mixed with honey was placed at the top of the trap. To allow ants to enter the trap, four holes were drilled on the wall of the tube. https://binary.pensoft.net/file/455301This dataset is made available under the Open Database License (http://opendatacommons.org/licenses/odbl/1.0/). The Open Database License (ODbL) is a license agreement intended to allow users to freely share, modify, and use this Dataset while maintaining this same freedom for others, provided that the original source and author(s) are credited.François Brassard, Chi-Man Leong, Hoi-Hou Chan, Benoit Guénard

Supplementary material 3 Figure S3. Schematic representation of ground nests used in the study Data type measurement Explanation note Four nests blocks, each containing two entrances of the same size (1.588, 1.984, 2.381 or 3.175 mm), were placed in a bundle. A nest bundle is represented with a top view in (A) and a sideview in (B). Details of the nest and an inside chamber are shown in (C). A zoomed in inset of a nest chamber containing a queen, a worker, a larva, eggs and pupae is shown in (D). For each site, 8 nests bundles (n = 64 nests) were placed. https://binary.pensoft.net/file/455302This dataset is made available under the Open Database License (http://opendatacommons.org/licenses/odbl/1.0/). The Open Database License (ODbL) is a license agreement intended to allow users to freely share, modify, and use this Dataset while maintaining this same freedom for others, provided that the original source and author(s) are credited.François Brassard, Chi-Man Leong, Hoi-Hou Chan, Benoit Guénard

Supplementary material 4 Figure S4. Transect design used for ground baiting Data type measurement Explanation note Each bait (n = 11) were placed 5 m apart along a 50 m transect. The baits, placed for 45 to 60 minutes, consisted of a 4 mm-thick slice of sausage previously dipped in honey. https://binary.pensoft.net/file/455303This dataset is made available under the Open Database License (http://opendatacommons.org/licenses/odbl/1.0/). The Open Database License (ODbL) is a license agreement intended to allow users to freely share, modify, and use this Dataset while maintaining this same freedom for others, provided that the original source and author(s) are credited.François Brassard, Chi-Man Leong, Hoi-Hou Chan, Benoit Guénard

Supplementary material 5 Table S5. List of the sampling sites visited in 2019, with their associated number, name and geolocation Data type species data Explanation note The date refers to the first sampling event made at a site, which corresponded to the leaf litter extraction and placement of subterranean traps. Sampling protocols are defined as follows: the letter (P) signifies a partial sampling protocol (i.e., leaf litter extraction, ground baiting and hand collection), whereas the letter (F) signifies a full protocol (i.e., leaf litter extraction, ground baiting, ground nests, subterranean traps, arboreal traps and hand collection). https://binary.pensoft.net/file/455304This dataset is made available under the Open Database License (http://opendatacommons.org/licenses/odbl/1.0/). The Open Database License (ODbL) is a license agreement intended to allow users to freely share, modify, and use this Dataset while maintaining this same freedom for others, provided that the original source and author(s) are credited.François Brassard, Chi-Man Leong, Hoi-Hou Chan, Benoit Guénard
