# Supplementary figures and images for: A new subterranean species and an updated checklist of Strumigenys (Hymenoptera, Formicidae) from Macao SAR, China, with a key to species of the Greater Bay Area
Source: Zookeys. 2020 Sep 21;970:63–116. doi: 10.3897/zookeys.970.54958 (PMC7578445; doi:10.3897/zookeys.970.54958)

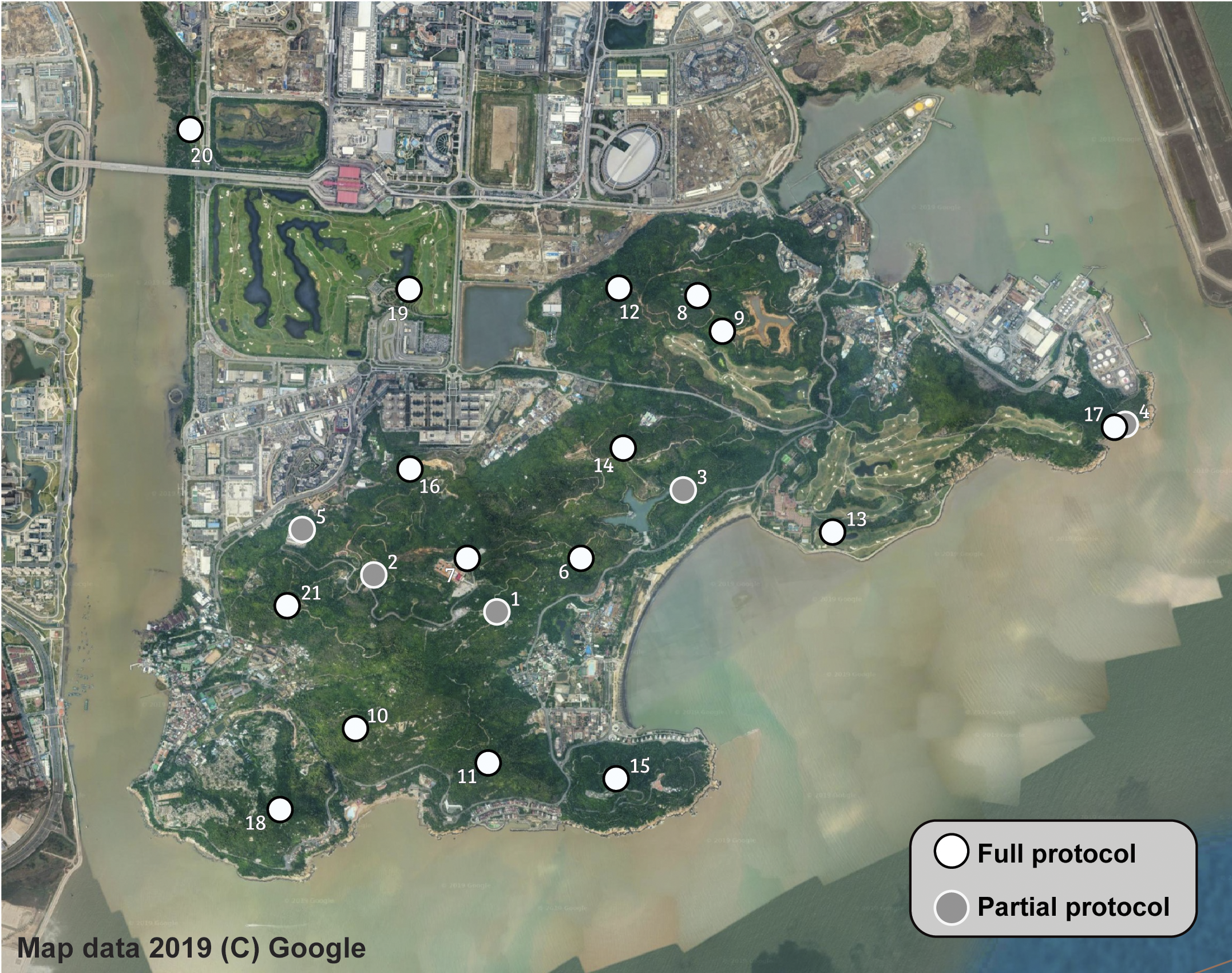

Supplement: Supplementary material 1 — Figure S1. Map of Coloane Island showcasing the 21 sites sampled [file zookeys-970-063-s001.tif]

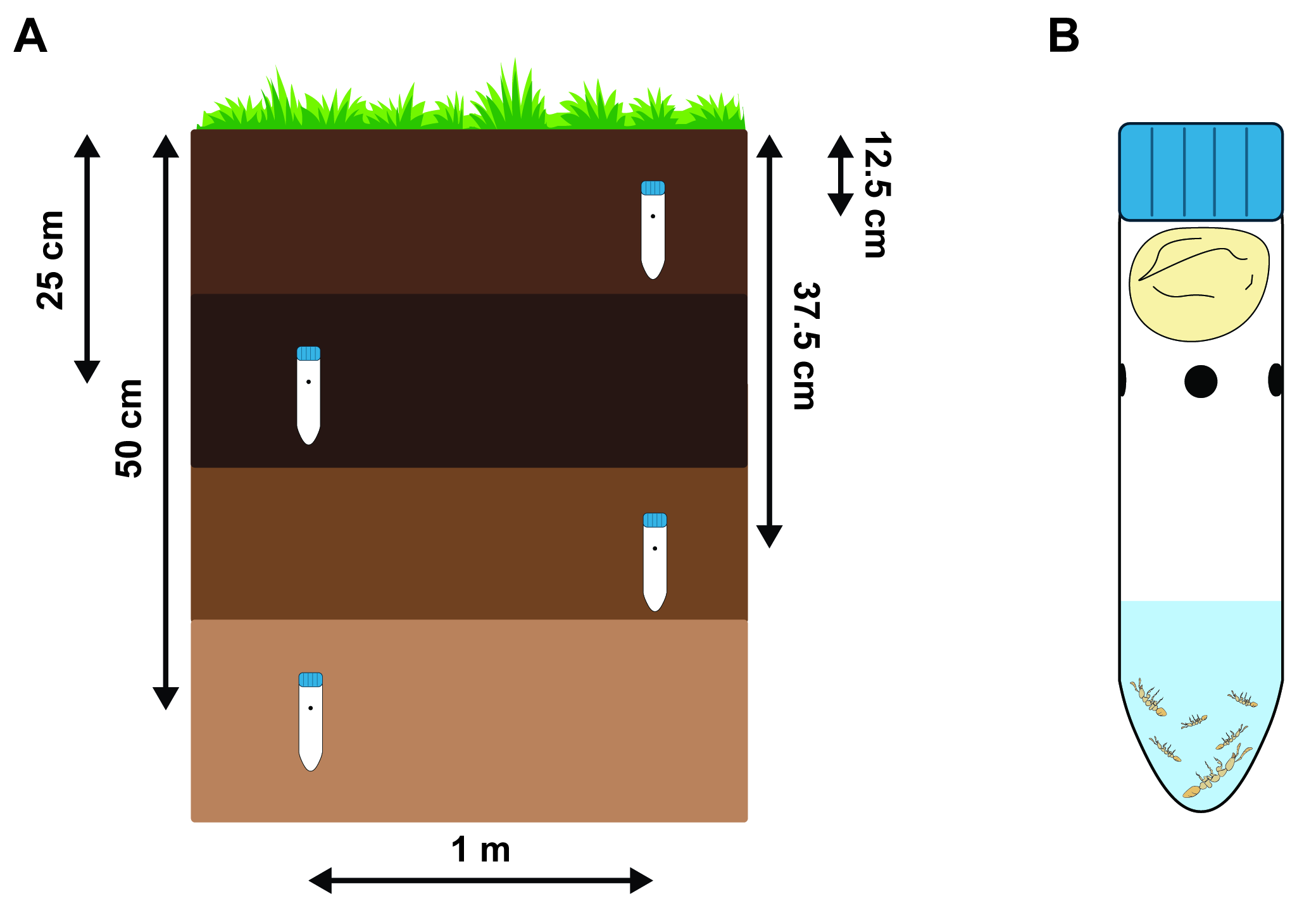

Supplement: Supplementary material 2 — Figure S2. Schematic representation of the subterranean sampling protocol used in the study [file zookeys-970-063-s002.tif]

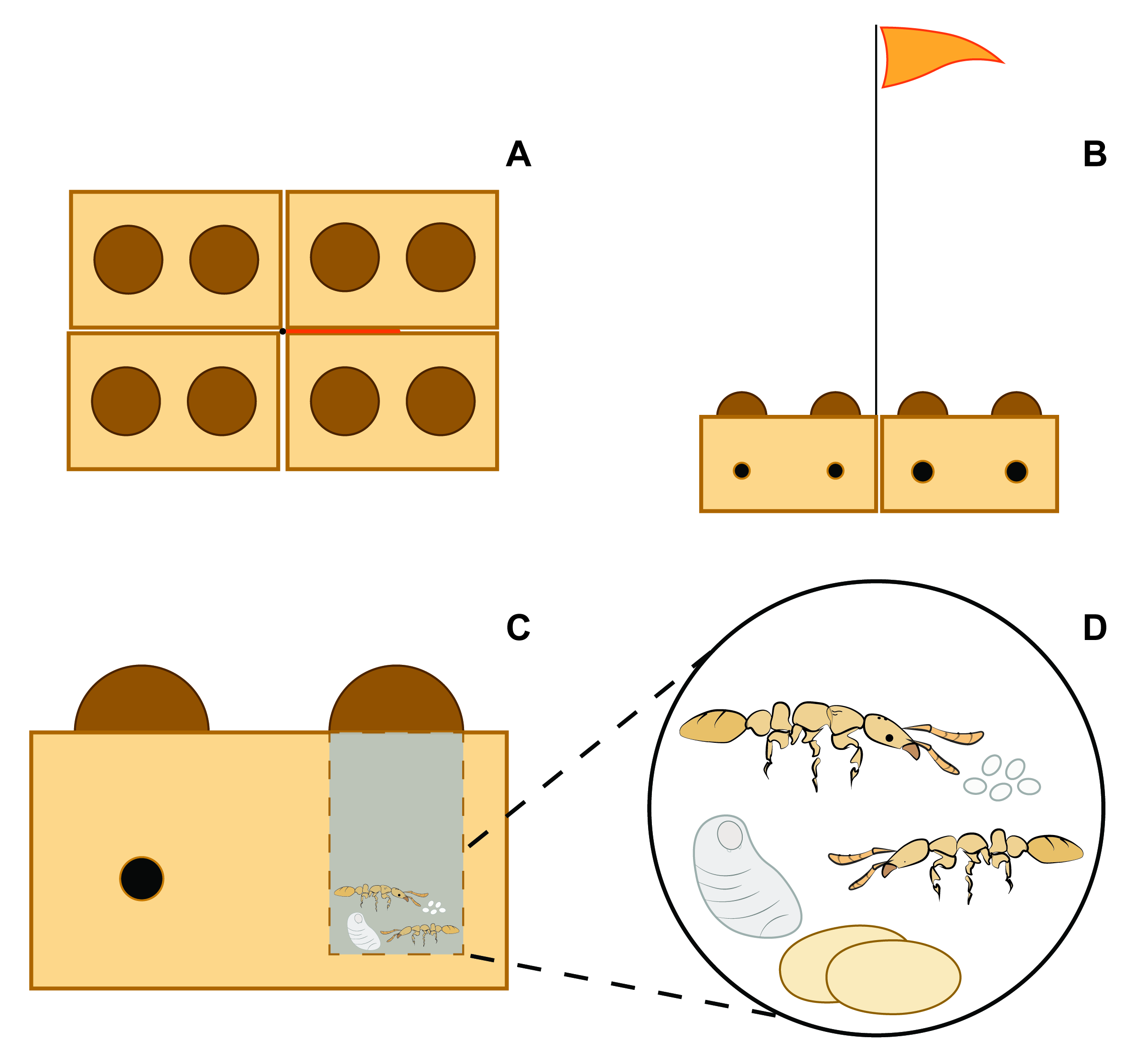

Supplement: Supplementary material 3 — Figure S3. Schematic representation of ground nests used in the study [file zookeys-970-063-s003.tif]

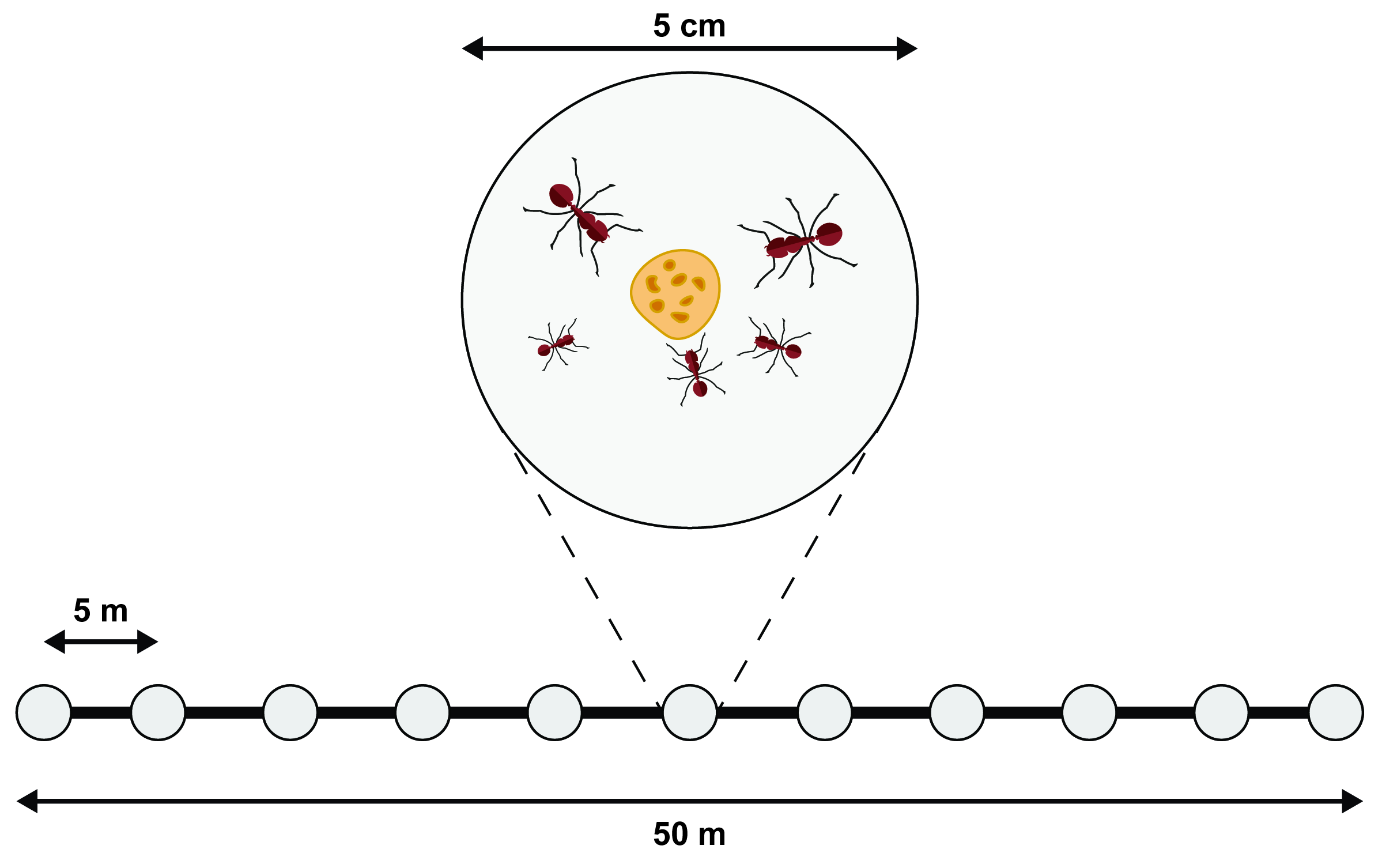

Supplement: Supplementary material 4 — Figure S4. Transect design used for ground baiting [file zookeys-970-063-s004.tif]
