# Supplementary material for: A new subterranean species and an updated checklist of Strumigenys (Hymenoptera, Formicidae) from Macao SAR, China, with a key to species of the Greater Bay Area
Source: Zookeys. 2020 Sep 21;970:63–116. doi: 10.3897/zookeys.970.54958 (PMC7578445; doi:10.3897/zookeys.970.54958)
Supplement: Supplementary material 5 — Table S5. List of the sampling sites visited in 2019, with their associated number, name and geolocation [file zookeys-970-063-s005.doc]

| **Site #** | **Site name** | **Latitude** | **Longitude** | **Elevation (m)** | **Date** | **Protocol** |
| --- | --- | --- | --- | --- | --- | --- |
| *1* | Coloane Trail (Path near C13 information point)  路環步行徑(C13定位點旁小徑) | 22.1214 | 113.5649 | ca. 110 | 18-III-2019 | P |
| *2* | Wetland in Alto de Coloane疊石谷濕地 | 22.1230 | 113.5597 | ca. 90 | 19-III-2019 | P |
| *3* | Hac Sá Reservoir Family Trail (Near C21 information point)  黑沙水庫家樂徑 (近C21定位點) | 22.1266 | 113.5727 | ca. 70 | 19-III-2019 | P |
| *4* | Ká-Hó Lighthouse 1  九澳燈塔1 | 22.1294 | 113.5914 | ca. 30 | 20-III-2019 | P |
| *5* | Seac Pai Van Park  石排灣郊野公園 | 22.1249 | 113.5566 | ca. 40 | 20-III-2019 | P |
| *6* | Hac Sá Reservoir Family Trail (Near 1-05-12 distance post)  黑沙水庫家樂徑 (近1-05-12標距柱) | 22.1237 | 113.5684 | ca. 90 | 8-IV-2019 | F |
| *7* | Estátua da Deusa A-ma  媽祖像 | 22.1237 | 113.5636 | ca. 160 | 8-IV-2019 | F |
| *8* | Ká-Hó Reservoir Freshwater Wetland  九澳水庫淡水濕地 | 22.1348 | 113.5733 | ca. 50 | 9-IV-2019 | F |
| *9* | Ká-Hó Reservoir Hillside  九澳水庫山邊 | 22.1333 | 113.5744 | ca. 90 | 9-IV-2019 | F |
| *10* | Coloane Trail (Near 1-01-10 distance post)  路環步行徑(近1-01-10標距柱) | 22.1165 | 113.5589 | ca. 100 | 10-IV-2019 | F |
| *11* | Coloane Trail (Near 1-01-15 distance post)  路環步行徑(近1-01-15標距柱) | 22.1151 | 113.5645 | ca. 80 | 11-IV-2019 | F |
| *12* | Coloane North East Hiking Trail  路環東北步行徑 | 22.1351 | 113.5700 | ca. 80 | 14-V-2019 | F |
| *13* | Macao Golf & Country Club澳門高爾夫球鄉村俱樂部 | 22.1248 | 113.5790 | ca. 20 | 15-V-2019 | F |
| *14* | Ká Hó Height Family Trail Peak (Near 1-09-03 distance post)  九澳高頂家樂徑 (近1-09-03標距柱) | 22.1284 | 113.5702 | ca. 140 | 16-V-2019 | F |
| *15* | Morro de Hac Sá Family Trail (Near 1-07-08 distance post)  黑沙龍爪角家樂徑 (近1-07-08標距柱) | 22.1144 | 113.5699 | ca. 50 | 17-V-2019 | F |
| *16* | Hillside of Department of Green Areas and Gardens  園林綠化廳後山 | 22.1275 | 113.5612 | ca. 70 | 20-V-2019 | F |
| *17* | Ká-Hó Lighthouse 2  九澳燈塔2 | 22.1292 | 113.5909 | ca. 30 | 21-V-2019 | F |
| *18* | Oscar Farm Hillside  金像農場後山 | 22.1131 | 113.5557 | ca. 80 | 24-VI-2019 | F |
| *19* | Caesars Golf Macau  澳門凱撒高爾夫 | 22.1351 | 113.5612 | ca. 10 | 25-VI-2019 | F |
| *20* | Cotai Ecological Zone II  路氹城生態保護區生態二區 | 22.1418 | 113.5519 | ca. 0 | 26-VI-2019 | F |
| *21* | Coloane Trail (Near C3 information point)  路環步行徑(近C3定位點) | 22.1217 | 113.5560 | ca. 110 | 27-VI-2019 | F |
